# Topical application of TAK1 inhibitor encapsulated by gelatin particle alleviates corneal neovascularization

**DOI:** 10.7150/thno.65098

**Published:** 2022-01-01

**Authors:** Jiang-Hui Wang, Ching-Li Tseng, Fan-Li Lin, Jinying Chen, Erh-Hsuan Hsieh, Suraj Lama, Yu-Fan Chuang, Satheesh Kumar, Linxin Zhu, Myra B. McGuinness, Jessika Hernandez, Leilei Tu, Peng-Yuan Wang, Guei-Sheung Liu

**Affiliations:** 1 Centre for Eye Research Australia, Royal Victorian Eye and Ear Hospital, East Melbourne, Australia.; 2 Graduate Institute of Biomedical Materials and Tissue Engineering, College of Biomedical Engineering, Taipei Medical University, Taipei, Taiwan.; 3 Menzies Institute for Medical Research, University of Tasmania, Hobart, Australia.; 4 Shenzhen Key Laboratory of Biomimetic Materials and Cellular Immunomodulation, Shenzhen Institute of Advanced Technology, Chinese Academy of Sciences, Shenzhen, China.; 5 Department of Ophthalmology, the First Affiliated Hospital of Jinan University, Guangzhou, China.; 6 Centre for Epidemiology and Biostatistics, Melbourne School of Population and Global Health, University of Melbourne, Melbourne, Australia.; 7 Ophthalmology, Department of Surgery, University of Melbourne, East Melbourne, Australia.; 8 Aier Eye Institute, Changsha, Hunan, China.

**Keywords:** anti-angiogenesis, corneal neovascularization (CoNV), transforming growth factor-β (TGF-β)-activated kinase 1 (TAK1), 5Z-7-oxozeaenol, gelatin nanoparticles (GNPs), eye drops

## Abstract

**Rationale:** Corneal neovascularization (CoNV) is a severe complication of various types of corneal diseases, that leads to permanent visual impairment. Current treatments for CoNV, such as steroids or anti-vascular endothelial growth factor agents, are argued over their therapeutic efficacy and adverse effects. Here, we demonstrate that transforming growth factor-β (TGF-β)-activated kinase 1 (TAK1) plays an important role in the pathogenesis of CoNV.

**Methods:** Angiogenic activities were assessed in *ex vivo* and *in vitro* models subjected to TAK1 inhibition by 5Z-7-oxozeaenol, a selective inhibitor of TAK1. RNA-Seq was used to examine pathways that could be potentially affected by TAK1 inhibition. A gelatin-nanoparticles-encapsulated 5Z-7-oxozeaenol was developed as the eyedrop to treat CoNV in a rodent model.

**Results:** We showed that 5Z-7-oxozeaenol reduced angiogenic processes through impeding cell proliferation. Transcriptome analysis suggested 5Z-7-oxozeaenol principally suppresses cell cycle and DNA replication, thereby restraining cell proliferation. In addition, inhibition of TAK1 by 5Z-7-oxozeaenol blocked TNFα-mediated NFκB signalling, and its downstream genes related to angiogenesis and inflammation. 5Z-7-oxozeaenol also ameliorated pro-angiogenic activity, including endothelial migration and tube formation. Furthermore, topical administration of the gelatin-nanoparticles-encapsulated 5Z-7-oxozeaenol led to significantly greater suppression of CoNV in a mouse model compared to the free form of 5Z-7-oxozeaenol, likely due to extended retention of 5Z-7-oxozeaenol in the cornea.

**Conclusion:** Our study shows the potential of TAK1 as a therapeutic target for pathological angiogenesis, and the gelatin nanoparticle coupled with 5Z-7-oxozeaenol as a promising new eyedrop administration model in treatment of CoNV.

## Introduction

Corneal neovascularization (CoNV) is a severe complication of various corneal diseases and the fourth leading cause of visual impairment worldwide 1. The avascular nature of the cornea, known as “angiogenic privilege”, is essential to maintain cornea transparency and optimal visual acuity 2. Pathological insults such as infection (viral, bacterial), inflammation (corneal graft rejection, Stevens-Johnson syndrome), hypoxia (incorrect contact lens use), trauma (mechanical trauma, chemical/thermal injury), corneal degeneration (loss of limbal barrier), or neoplasm can promote the invasion of new vessels sprouting from capillaries/venules of pericorneal plexus into the avascular cornea, causing CoNV 2, 3. With CoNV, corneal transparency is compromised, blocking light entry. The increased vascular permeability of these new abnormal vessels also induces a cycle of inflammation, chronic edema, lipid exudation, and corneal scarring, potentially resulting in permanent vision impairment 4. Cornea transplantation (keratoplasty) is the most obvious solution for CoNV. However, it has been reported that existing neovascularization at the border of the cornea may rapidly invade the new graft, increasing the risk of rejection, and limiting clinical utility of keratoplasty 5. For relatively mature CoNV, surgical therapies with physical vessel ablation using fine needle diathermy, laser, or photodynamic therapy (PDT) are typically employed 2.

The current pharmacotherapies for CoNV include topical steroid and anti-vascular endothelium growth factor (VEGF) agents. However, steroid treatment is limited to early stage of CoNV with a narrow temporal window due to its limited angioregressive capacity 2, 6. Furthermore, long-term use of steroids raises a multitude of side effects such as elevated intraocular pressure, increased risk of infection, and posterior subcapsular cataracts 7. Targeted molecular therapies in CoNV, especially the off-label use of anti-VEGF drugs, has gained interest recently due to its success in treating choroidal and retinal NV 8. Commercially available anti-VEGF drugs including aflibercept, ranibizumab, and bevacizumab have shown promising results in treating CoNV 9. However, partial efficacy and adverse effects, such as reduced epithelial healing and corneal thinning, have been reported 10. Similar to steroids, the efficacy of anti-VEGF drugs in mature CoNV is still controversial 3, 11, 12. Thus, alternative therapeutic approaches that are safer and more effective are needed to improve the treatment options for CoNV.

Transforming growth factor-β (TGF-β)-activated kinase 1 (TAK1), a serine/threonine kinase, is a master regulator for a wide range of physiological and pathological cellular processes 13, 14. TAK1 can be activated by multiple stimuli including interleukin-1 (IL-1), tumor necrosis factor α (TNFα), TGF-β, or toll-like receptor (TLR) ligands, and predominantly modulate the cell death and inflammatory signalling pathways 15, 16. TAK1 was found to support vascular formation and endothelial migration. A study showed that pharmacological inhibition of TAK1 by 5Z-7-oxozeaenol provided a potential therapeutic effect on pathological retinal angiogenesis by modulating inflammatory and angiogenic signalling 17, 18. Indeed, constant TAK1 expression protects against endothelial cell apoptosis beyond inflammatory conditions, and genetic TAK1 knockout leads to embryonic lethality due to endothelium death and deleterious vasculature development 19. The therapeutic roles of TAK1 in ocular angiogenesis leads to the present study to investigate if TAK1 can be a potential anti-angiogenesis target in CoNV.

We hypothesized TAK1 to play a crucial role in pathological angiogenesis and that TAK1 inhibition can suppress CoNV. Since the ideal therapeutic strategy for CoNV requires drugs that can be delivered to the ocular surface topically for long-term inhibition of angiogenesis, we have developed a novel eye drop formulation based on gelatin nanoparticle (GNPs) to encapsulate a selective TAK1 inhibitor, 5Z-7-oxozeaenol. Gelatin, an FDA-approved biomaterial, is especially suitable for treating corneal disease. It is a collagen-based biopolymer, which enhances interaction with collagen-enriched corneal stroma layer when delivering drugs using GNPs 20. In this study, we evaluated the anti-angiogenic activities of 5Z-7-oxozeaenol in human endothelial cells. Moreover, a mouse model of chemical cauterization-induced CoNV was used to investigate the therapeutic effect and underlying mechanisms of GNPs-encapsulated 5Z-7-oxozeaenol (GNPs-Oxo) via eye drop administration.

## Materials and Methods

### Aortic ring assay

Aortic ring assay was performed as previously described 21. Briefly, aortae were removed from euthanized C57BL/6 mice and immediately transferred to a culture dish containing ice-cold EBM^TM^ plus basal medium supplied with EGM^TM^ Plus SingleQuots^TM^ supplements (CC-5035; Lonza, Basel, Switzerland). The peripheral fibroadipose tissue was carefully removed. The aortae were sectioned into rings ~1 mm in width. Each aortic ring was placed in an individual well covered with 120 μL Matrigel™ Basement Membrane Matrix (356234; Corning, New York, NY) and was immersed in 200 μL complete medium with or without 1 µM 5Z-7-oxozeaenol (3604/1; Tocris Bioscience, Bristol, UK) for 9 days in a 48-well plate. The medium was refreshed, and 5Z-7-oxozeaenol was re-supplemented every 3 days. Images of individual explants were taken from day 0 to day 9. The neo-formed vascular sprouting area was quantified with Adobe Photoshop Elements 13.

### Cell culture

The telomerase-immortalized human microvascular endothelium (TIME) cell line was purchased from American Type Culture Collection (CRL-4025; ATCC, Manassas, VA). Endothelial cells were maintained in EBM^TM^ plus basal medium supplied with EGM^TM^ Plus SingleQuots^TM^ supplements. Cells were maintained in a humidified incubator at 37 °C and 5% CO_2_. Cells were tested for mycoplasma using MycoAlert™ Mycoplasma Detection Kit (LT07; Lonza).

### Cell proliferation assay

CYQUANT^®^ NF cell proliferation assay kit was used to detect cell proliferation according to the manufacturer's instructions (C35006; Life Technologies Australia, Mulgrave, VIC, Australia). In brief, endothelial cells were seeded at the density of 5 × 10^3^ per well in 96-well plate. Cells were pre-treated with 1 μM 5Z-7-oxozeaenol or vehicle. The conditioned medium was removed 24 or 48 hours after drug exposure and replaced with DNA binding solution. The plate was incubated at 37 °C for 1 hour followed by the assessment of fluorescence intensity. The fluorescence intensity was measured by a Spark^®^ Multimode Microplate Reader (Tecan; Männedorf, Switzerland) with excitation at 485 nm and emission detection at 530 nm. Results were normalized to percentage of control.

### RNA isolation

Total cellular or tissue RNA was isolated using Zymo Quick-RNA MiniPrep kit (R1055; Zymo Research, Irvine, CA) according to the manufacturer's instructions. The quantity of RNA was evaluated with a NanoDrop ND-1000 spectrophotometer (NanoDrop Technologies, Wilmington, DE).

### RNA-seq analysis

Analysis was undertaken in triplicates on total RNAs (1 μg) from TIME cells treated with 0.2 μM or 1 μM 5Z-7-oxozeaenol or vehicle prepared as per the manufacturers' instructions. cDNA libraries were sequenced (50 bp pair end reads) by GENEWIZ (Suzhou, China) using an Illumina Novaseq PE150 platform. The adapter sequences were removed from the raw fastq files, and the low-quality reads were dropped by Trimgalore v0.4.4 (Babraham Bioinformatics). Filtering parameters were set as follow: -q 25 --length 50 -e 0.1 --stringency 5. The trimmed reads were subjected to alignment using STAR v2.5.3, with default settings, against human reference genome hg38. Aligned RNA-seq data was counted over gene exon using featureCounts (subread 1.6.4). Genes were annotated as per the Gencode Version 33 annotation file.

### Gene set enrichment analysis (GSEA)

GSEA was performed with RNA-seq data (0.2 µM 5Z-7-oxozeaenol vs. control and 1 µM 5Z-7-oxozeaenol vs. control) against canonical pathway genesets collection (c2.cp.v7.2, Broad Institute) using 1000 genesets permutations. Only genesets with FDR < 0.05 were considered. Readcount values were loaded into R (v3.6.3) for statistical analysis and the pheatmap and gochord function was used to perform hierarchical clustering analysis and draw chord plot, respectively for data representation.

### Western blotting

Protein was extracted from cells or retinas with Pierce RIPA buffer (89900; Life Technologies Australia) supplied with protease inhibitor cocktail (11697498001; Roche Diagnostics, Basel, Switzerland). Equal amounts of cell lysates from different groups were subjected to Western blotting analysis with specific antibodies against IκB, phospho-NFκB p65, NFκB p65 (4814, 3033, 4764, respectively; Cell Signaling Technology, Danvers, MA), and β-actin (MAB1501; Merck Millipore, Burlington, MA). The quantitative densitometry was analyzed using Image Pro Plus software (Media Cybernetics, Rockville, MA).

### Quantitative polymerase chain reaction (qPCR)

Total RNA (100 ng) was reverse transcribed to cDNA using a High-Capacity cDNA Reverse Transcription kit (4368814; Life Technologies Australia). Quantitative PCR was performed using TaqMan fast advanced master mix (4444553; Applied Biosystems, Foster City, CA) and TaqMan probe sets to detect gene expression (**Table S1**). The expression levels of target genes were normalized to human GAPDH levels (Hs99999905_m1; Applied Biosystems). Subsequently, the ∆∆Ct method was used to evaluate relative expression level (fold change) in each condition versus the corresponding control condition.

### Cell migration assay

TIME cells were seeded at the density of 4 x 10^4^ cells per well in a 96-well plate. The wound to the cell monolayers were made by using an Incucyte® 96-well WoundMaker Tool (4563; Incucyte, Ann Arbor, MI) to create an incision-like gap on the following day. Cells were then exposed to 5Z-7-oxozeaenol (1 µM) with or without TNFα (10 ng/mL) (PHC3015; Life Technologies Australia) in 100 µl EGM^TM^ Plus basal medium containing VEGF (20 ng/mL) (293-VE-010; R&D Systems, Minneapolis, MN). The scratch area was photographed using an Incucyte® Live-Cell Imaging and Analysis System (Incucyte) immediately after wounding (0 hour) and 16 hours post-wounding. The scratch area was quantified using ImageJ v1.48 software (http://imagej.nih.gov/ij/). Cell migration was expressed as a percentage of closure of the scratch area: (scratch area at 0 hour - scratch area at 16 hour) / scratch area at 0 hour × 100%.

### Endothelial tube formation assay

Endothelial tube formation was processed as previously described 22. TIME cells were seeded at a density of 2 × 10^5^ cells/well in a 6-well plate. Cells were then pre-treated with 5Z-7-oxozeaenol (1 µM) for 30 minutes followed by TNFα (10 ng/mL) treatment for 24 hours. Matrigel™ Basement Membrane Matrix was thawed at 4°C overnight and coated evenly over each well (50 μL) in a 96-well plate. The plate was then incubated at 37°C for 30 minutes to allow Matrigel polymerization. Conditioned endothelial cells were trypsinized and then seeded at the density of 3 × 10^4^ cells per well in 100 μL EGM^TM^ Plus basal medium containing VEGF (20 ng/mL). Each condition was tested with three replicates and images were taken 6 hours post treatment. Images were photographed using an Incucyte® Live-Cell Imaging and Analysis System at defined time points. Endothelial mesh numbers were quantified by the ImageJ (version 1.48) installed with Angiogenesis Analyzer Plugin.

### Nanoparticle Preparation

The gelatin nanoparticles (GNPs) were prepared by a two-step desolvation method as previously described with modifications 23. Type A gelatin (bloom 175) (G2625; Sigma-Aldrich, St. Louis, MO) was used and purified by acetone (32201; Sigma-Aldrich) for first desolvation, followed by dissolution with hot water to get the final concentration of 1% (w/v) with the adjusted pH value of 2.5. 5Z-7-oxozeaenol was first dissolved in Dimethyl Sulfoxide (DMSO) (9224-01; JT Baker, Phillipsburg, NJ) at concentration of 2.5 mg/mL. 50 μL 5Z-7-oxozeaenol solution was mixed with 1 mL gelatin solution. Acetone was dropwise added again to form nanoparticles. Thereafter, 50 μL glutaraldehyde solution (8%, v/v) (G6257; Sigma-Aldrich), as crosslinking agent to stabilize GNPs, was added and stirred for 3 hours at 1000 rpm. The remaining organic solvent was evaporated using a rotary evaporator (EYELA; Japan). These 5Z-7-oxozeaenol-loaded GNPs were stored at 4°C for following studies.

### Characterization of gelatin nanoparticle-encapsulated 5Z-7-oxozeaenol

After preparation of GNPs-Oxo, its particle size and zeta potential were examined by the dynamic light scattering (DLS) analyzer (Zetasizer Nano ZS90 Plus; Malvern Panalytical, Malvern, UK) at 25 °C, with scattering light at 90 degrees and 180 s. Particle polydispersity index (PDI) was recorded by the same equipment. The morphology of GNPs-Oxo was observed by a HT-7700 transmission electron microscope (TEM) (Hitachi, Japan) at acceleration voltage of 80.0 kV. For TEM sample preparation, the gelatin nanoparticle was dropped onto carbon coated nickel 300 mesh (Ted Pella Inc.; Redding, CA) with subsequent staining of 0.5% uranium acetate (541-09-3; Thermo Fisher Scientific, Waltham, MA). Nanoparticle Tracking Analysis (NTA) (NanoSight NS300; Malvern Panalytical, UK) was further used to observe particles and to count for concentration under liquid form. The colloidal solution with nanoparticle was examined with laser at 565 nm wavelength at room temperature, and viscosity was set with 1.0cP (water). GNPs and GNPs-Oxo were examined after dilution, and results were analyzed in NanoSight NTA 3.4 Build 3.4.003 software. The samples were measured for 45 seconds with 1200 shutter and 146 gain alignments, which provided a clearer view of NP for tracking by video. The 5Z-7-oxozeaenol concentration was quantified using high performance liquid chromatography (HPLC, Merck Hitachi LaChrome, Tokyo, Japan). The Eclipse plus C18 column (5 μm, ZORBAX; Agilent Technologies, Santa Clara, CA) and the mobile phase included acetonitrile (AE0627; Aencore Chemical, Surrey Hills, VIC, Australia)/water in the volume ratio of 2:3 was used. The flow rate of mobile phase was set as 1 mL/min. 5Z-7-oxozeaenol was then identified by UV at the wavelength of 350 nm. A series concentration of 5Z-7-oxozeaenol ranging from 1 to 50 μg/mL was prepared and measured as a standard curve. The drug encapsulation efficiency (EE) is calculated as the final 5Z-7-oxozeaenol content compared with the initial amount of 5Z-7-oxozeaenol addition; the drug loading efficiency (LE) is calculated as the ratio of final 5Z-7-oxozeaenol content in the whole gelatin base.

### Drug release assessment *in vitro*

Dialysis method was used to study the drug release efficacy of 5Z-7-oxozeaenol or GNPs-Oxo. Briefly, 1 mL of GNPs-Oxo or 5Z-7-oxozeaenol in PBS (pH 7) at the same concentration (750 mg/mL) were transferred to dialysis bags (Spectra/Por®, Float-A-Lyzer® G2 Dialysis Device, MWCO 20 kDa, Z726710-12EA; Sigma-Aldrich), which was immersed in 15 mL of release buffer (15% ethanol) and centrifuged at 50 rpm at 37 °C. 300 μL of release buffer was sampled at 0, 0.2, 0.5, 1, 3, 6, 12, 24, 48 hours, and was replaced with an equal volume of fresh release buffer. The sample buffer was filtrated through a 0.22 μm membrane filter, and the concentration of 5Z-7-oxozeaenol in samples at different time points was determined by HPLC as previously described.

### Animals and ethics statement

Male mice (C57BL/6J, 8 to 14 weeks old) were used in this study. Animals were housed in standard cages with a temperature/humidity-controlled and a 12-hour light (50 lux illumination)/12-hour dark (<10 lux illumination) cycle environment. Food and water were available *ad libitum*. The use of animal was conducted in strict accordance with the Association for Research in Vision and Ophthalmology (ARVO) statement for ophthalmic and vision research and approved by the Institutional Animal Care and Use Committee (IACUC) of Taipei Medical University (LAC-2017-0344 and LAC-2019-0613).

### Assessment of intracellular distribution of nanoparticles and its retention on the corneal surface

The intracellular distribution of nanoparticles was examined in human umbilical vein endothelial cells (HUVECs, C0035C; Life Technologies Australia). HUVECs were seeded in 96 well-plates at 5×10^3^ cells/well prior to treatment. The 5Z-7-oxozeaenol, GNPs, and GNPs-Oxo solution conjugated with 5-Carboxytetramethylrhodamine succinimidyl ester (TAMRA) (C2211; Thermo Fisher Scientific) or TAMRA dye alone (1 μg/mL) were added in the medium to co-culture with HUVECs for 0.5 and 2 hours. Cells were washed by PBS for 3 times and fixed by 4% paraformaldehyde for 15 minutes and washed by PBS for several times at each time point, followed by DAPI staining for 10 minutes. Finally, cells were examined by ImageXpress Pico automatic cell imaging system (Molecular Devices, CA) under a 20X objective lens at wavelength of Ex/Em: 359 nm/461 nm for DAPI (1:50 dilution, stock 10 mg/mL; Generon, Slough, UK) and Ex/Em: 546 nm/579 nm for TAMRA. Images were analyzed by View Analyze software.

C57BL/6 mice were live monitored using an IVIS-200 *in vivo* imaging system (Xenogen Corporation; San Francisco, CA) to access the retention of fluorescent NPs on the ocular surface. The TAMRA dye was conjugated to the GNPs and GNPs-Oxo. The TAMRA-conjugation protocol was previously described 24. To prepare eye drop formulation, TAMRA solution (as free-form fluorescent dye), TAMRA-conjugated GNPs and GNPs-Oxo were diluted with PBS and adjusted to same TAMRA concentration before use. Following anesthesia by an intraperitoneal injection of a mixture of zolazepam and tiletamine (Zoletil 50^®^, Virbac; France) and xylazine (Rompun^®^, Bayer; Korea), mice were administered with 5 μL eye drop per eye and photographed for ocular fluorescence at multiple time points. The subtraction of tissue autofluorescence was performed to eliminate background noise to improve sensitivity. Nine mice were used in this experiment (n = 3 per group; free dye, GNPs, and GNPs-Oxo).

A similar test for topical delivery of nanoparticles to mouse eyes was performed as follows: the free dye and dye conjugated GNPs/GNPs-Oxo were adjusted in the same fluorescence signalling intensity. Mice were administered with 5 μL eye drop per eye and waited for 2 hours. The entire eyeball of each mouse was enucleated, soaked in modified Davidson's fluid (CIS-Bio, Taipei, Taiwan) for 24 hrs, followed by fixation with 10% neutral buffered formalin solution (CIS-Bio). The eyeballs were embedded in frozen section media (Leica, Wetzlar, Germany) for cryosection. A cryostat microtome (CM 3050S; Leica) was used to section the frozen specimens. The cryosections were washed by PBS twice to remove the frozen media, followed by staining with DAPI for 30 minutes. The cryosections were de-stained several times by PBS washing before mounting with the mounting medium (F4680; Sigma-Aldrich). A laser confocal microscope (TCS SP5 Confocal spectral microscope imaging system; Leica) was used to examine nanoparticle distribution in the cornea. Nine mice were used in this experiment (n = 3 per group; free dye, GNPs, and GNPs-Oxo).

### Corneal model of neovascularization and assessment of corneal neovascularization

Mouse model of CoNV was established according to previous protocols 25, 26. Briefly, the mice were first anesthetized with mixture of Zoletil 50^®^ and Rompun^®^, followed by the topical administration of 0.5% Alcaine® (Alcon; Geneva, Switzerland) for local anesthesia. The chemical cauterization-induced CoNV was conducted by pressing the 75% silver nitrate/25% potassium nitrate applicator (1590; Grafco, Australia) to the center of cornea steadily for eight seconds, with subsequent saline flushing to remove excess of nitrate. Only one eye of each mouse was chemically burnt. To prepare eye drops, 5Z-7-oxozeaenol, GNPs or GNPs-Oxo solution was diluted with PBS to achieve final 5Z-7-oxozeaenol concentration of 5 µg/mL and gelatin concentration of 700 µg/mL. 5 µL of eye drop was applied to mouse eye once daily, for 7 days. Induced CoNV was characterized by vessels sprouting from limbal vasculature towards the burnt central cornea 27. The severity of CoNV and burn stimulus response were evaluated using a SL-17 hand-held portable slit lamp (Kowa Company Ltd.; Japan) and imaged. The extent of CoNV was graded from 0 to 6 according to the extent of vessel growth from limbus to burnt edge as following: 0: no visible vessels; 1: 1/4 distance to edge of burn; 2: 1/3 distance to edge of burn; 3: 1/2 distance to edge of burn; 4: 2/3 distance to edge of burn; 5: 3/4 distance to edge of burn; 6: vessels reach edge of burn) 25. For vessel quantification, the total area (from limbus to conjunctiva-surface layer of sclera) and vessel area were manually selected with ImageJ. The vessel area was presented as the percentage of the total area with the following formula: (selected vessel area / total area counted) x 100%. Seventy mice were used in this study for three repeated tests (n=14 per group; normal, PBS, GNPs, 5Z-7-oxozeaenol, and GNPs-Oxo).

### Statistical analysis

All data are expressed as mean ± SEM from at least three independent experiments. Statistical differences were obtained by performing Student's t-test, one-way or two-way ANOVA wherever appropriate followed by Tukey's multiple comparisons test using GraphPad Prism 7 (GraphPad; San Diego, CA). A piece-wise linear mixed-effects model with a single knot at 10 minutes was used to assess the rate of decay in fluorescent intensity over time in **Figure 4F**. P-values less than 0.05 were considered statistically significant.

## Results

### TAK1 inhibitor, 5Z-7-oxozeaenol, suppresses endothelial proliferation via impaired the cell cycle and DNA replication pathway

**Figure 1:**
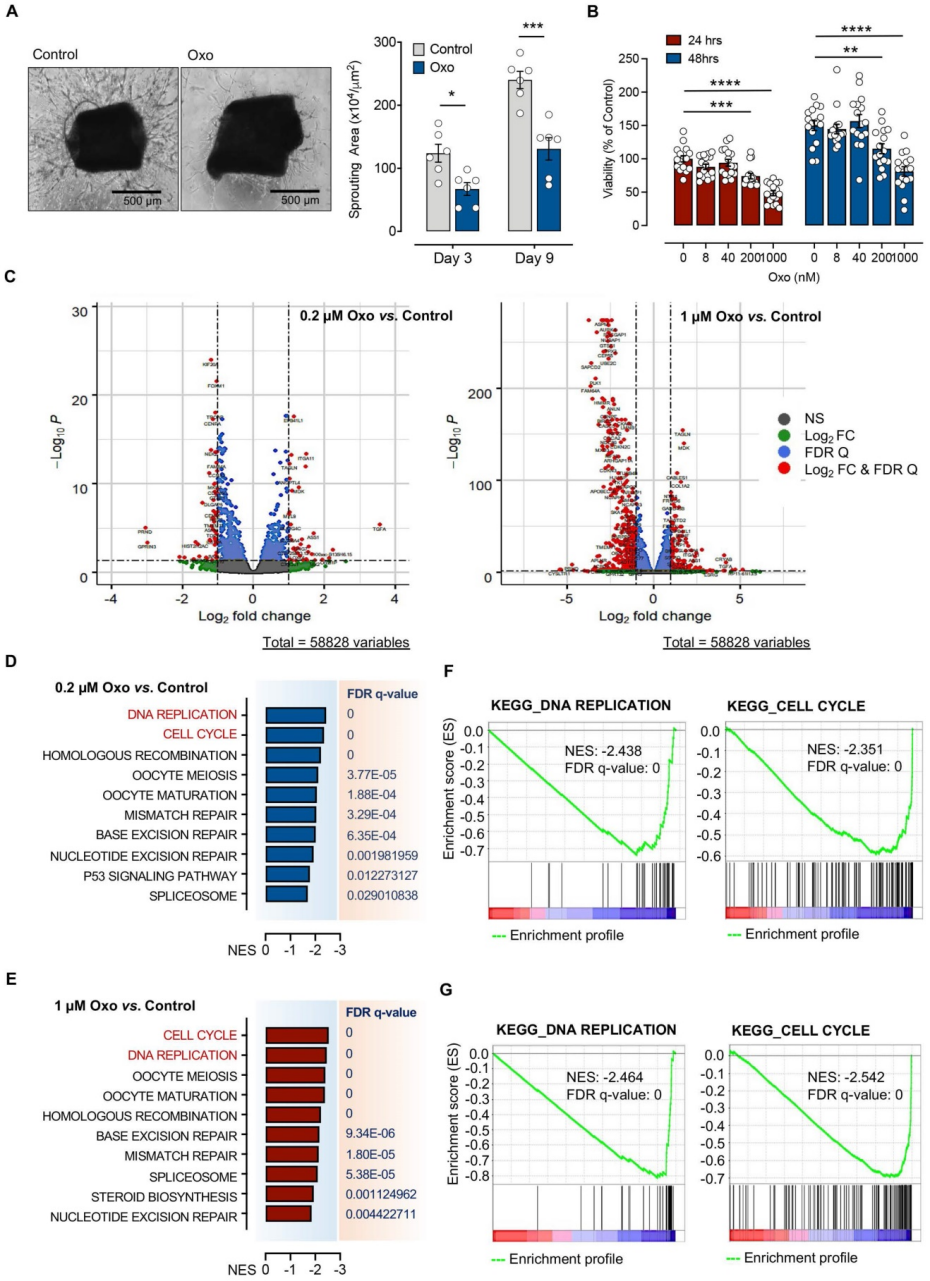
** Pharmacologic inhibition of TAK1 by 5Z-7-oxozeaenol suppressed angiogenic activities *in vitro* and* ex vivo* by inhibiting cell cycle and DNA replication*.* (A)** Sprouting assay was performed in sectioned mouse aortic rings in the presence or absence of the TAK1 inhibitor, 5Z-7-Oxozeaenol (Oxo), and the sprout area was quantified 3 and 9 days after 5Z-7-oxozeaenol (Oxo) treatment (n = 6 from three independent experiments). Representative images were taken at day 9. Scale bar: 500 ìm. **(B)** Human telomerase-immortalized human microvascular endothelial (TIMEs) cells were pre-treated with delivery vehicle or 5Z-7-Oxozeaenol (Oxo) at 8, 40, 200 or 1000 nM for 24 or 48 hours, followed by the cell viability assay (n = 16 from four experiments). **(C)** RNA-Seq was performed for TIME cells treated with 0.2 µM or 1 µM 5Z-7-oxozeaenol (Oxo) or vehicles for 24 hours, followed by bioinformatic analysis (n = 4 independent biological replicates). Volcano plots show that level of gene expression was significantly changed in 0.2 µM or 1µM 5Z-7-oxozeaenol (Oxo)-treated TIME cells. Red dots refer to genes showing a Log_2_(fold change) > 1 or < -1 and False discovery rate (FDR) < 0.05. **(D-E)** GSEA indicated that several gene sets (Reactome Pathway Database) for canonical pathways were negatively enriched in 0.2 µM- or 1 µM 5Z-7-oxozeaenol (Oxo)-treated TIME cells. **(F-G)** GSEA plots showing negative association with the 'DNA Replication' and 'Cell Cycle' gene sets in 0.2 µM or 1 µM 5Z-7-oxozeaenol (Oxo)-treated TIME cells compared to controls (vehicles). Statistical analysis was conducted by two-way RM ANOVA and Tukey's multiple comparison test; *P < 0.05, **P < 0.01, ****P < 0.0001.

5Z-7-oxozeaenol, a resorcylic acid lactone derived from a fungus, is a selective inhibitor of TAK1 28. We first assessed the effect of 5Z-7-oxozeaenol on endothelial cell sprouting *ex vivo* and proliferation *in vitro*, both are important factors in the development of pathological angiogenesis. Aortic ring assays revealed that 1µM 5Z-7-oxozeaenol treatment resulted in great reduction of tube sprouting from the aortic ring explant at both observation timepoints: day 3 (45.9% reduction; *P* < 0.05) and day 9 (45.8% reduction; *P* < 0.001), further indicating the inhibitory effects of 5Z-7-oxozeaenol on cell proliferation (**Figure 1A**). Furthermore, 5Z-7-oxozeaenol showed a significant inhibitory effect on TIMEs proliferation for 24 and 48 hours in a dose-dependent manner (p<0.01; **Figure 1B**).

**Figure 2:**
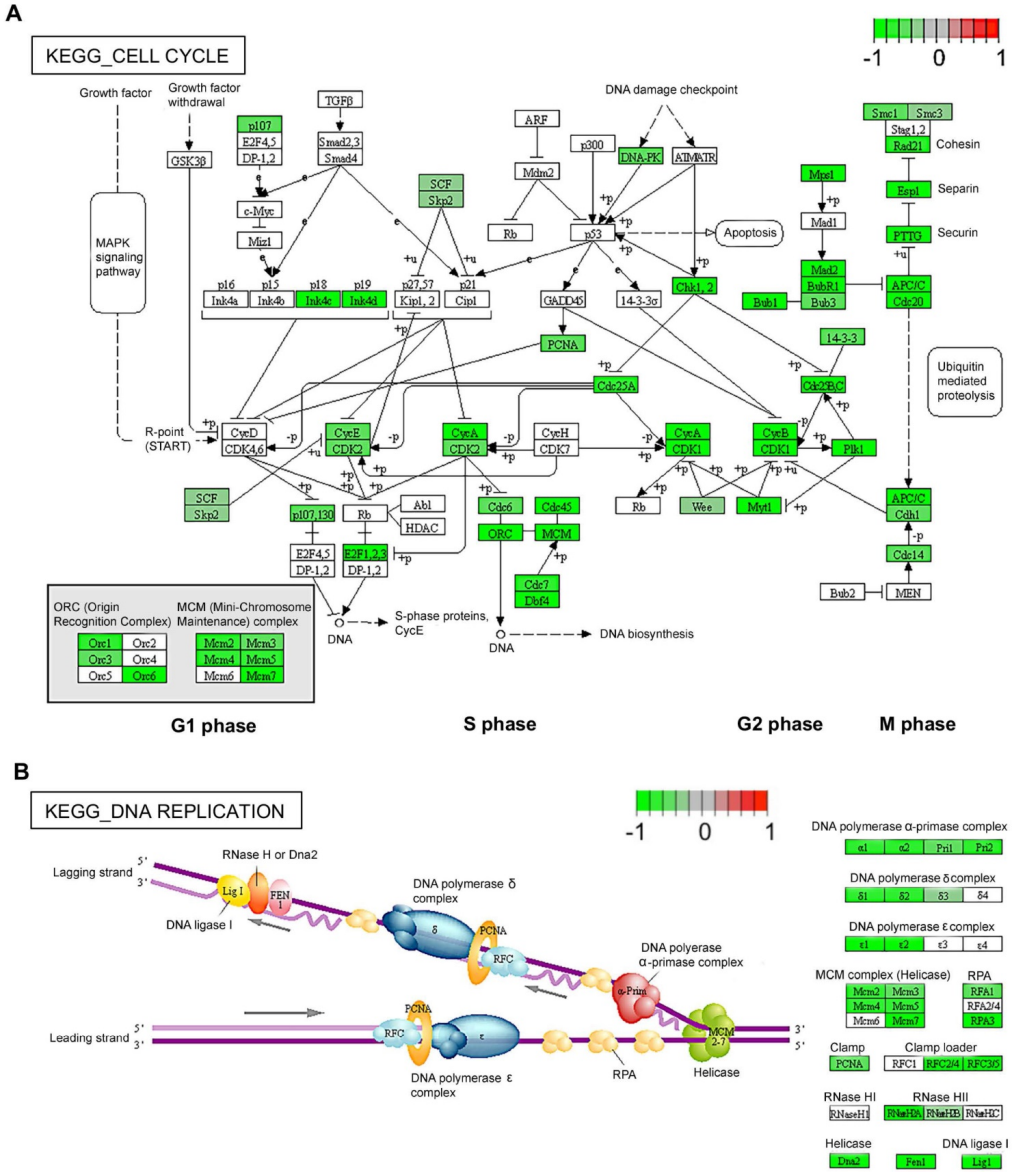
** Diagrams for two significantly changed KEGG pathways plotted using Pathview 64. (A)** Cell cycle.** (B)** DNA replication. Intensity of colour represents gene Log_2_ (fold change) with downregulation in green and upregulation in red. Pathway maps are displayed with copyright permission from KEGG.

To understand the role of TAK1 inhibition in cell proliferation, we performed bulk RNA-sequencing (RNAseq) for TIME cells treated with 0.2 µM or 1 µM 5Z-7-oxozeaenol (**Figure S1A**). Further transcriptome analysis revealed that a significant number of genes was dysregulated in cells treated with both doses of 5Z-7-oxozeaenol (699 and 4223 dysregulated genes found in 0.2 µM and 1 µM 5Z-7-oxozeaenol, respectively; **Figure 1C; Figure S1B, Supplementary databases 1 and 2**). To identify the key molecular signalling or biological pathways relevant to the inhibitory effects of 5Z-7-oxozeaenol in TIME cells, the transcriptome dataset was interrogated by taking advantage of Gene Set Enrichment Analysis (GSEA), a knowledge-based approach for interpreting genome-wide expression profiles. GSEA-based analysis revealed negative enrichment of several signalling pathways in the cells treated with two different doses of 5Z-7-oxozeaenol. When comparing 5Z-7-oxozeaenol with control, the analysis confirmed the downregulation of gene sets that are representative of several biological pathways relevant to TAK1 inhibition (**Figure 1D-E**). Among others, “DNA Replication” and “Cell Cycle” topped the list for cells treated with two different doses of 5Z-7-oxozeaenol, suggesting that TAK1 inhibitory effects on cell proliferation impedes these pathways (**Figure 1F-G; Figure S2, S3, Supplementary databases 3 and 4**). To clearly view the dysregulated genes in the affected pathways, we plotted the KEGG pathway map, including cell cycle (PATHWAY: map04110) and DNA replication (PATHWAY: map03030). As shown in the KEGG pathway map (**Figure 2A**), all the dysregulated genes in the “Cell Cycle” pathway were downregulated and involved in all stages of the cell cycle. Likewise, all dysregulated genes in the “DNA Replication” pathway were downregulated (**Figure 2B**). A circular plot (**Figure S4A**) summarized all down-regulated genes involved in the two pathways, and the normalized expression of each gene (**Figure S4B-C**).

### 5Z-7-oxozeaenol suppresses cytokine-mediated inflammatory signalling and angiogenic activities

**Figure 3:**
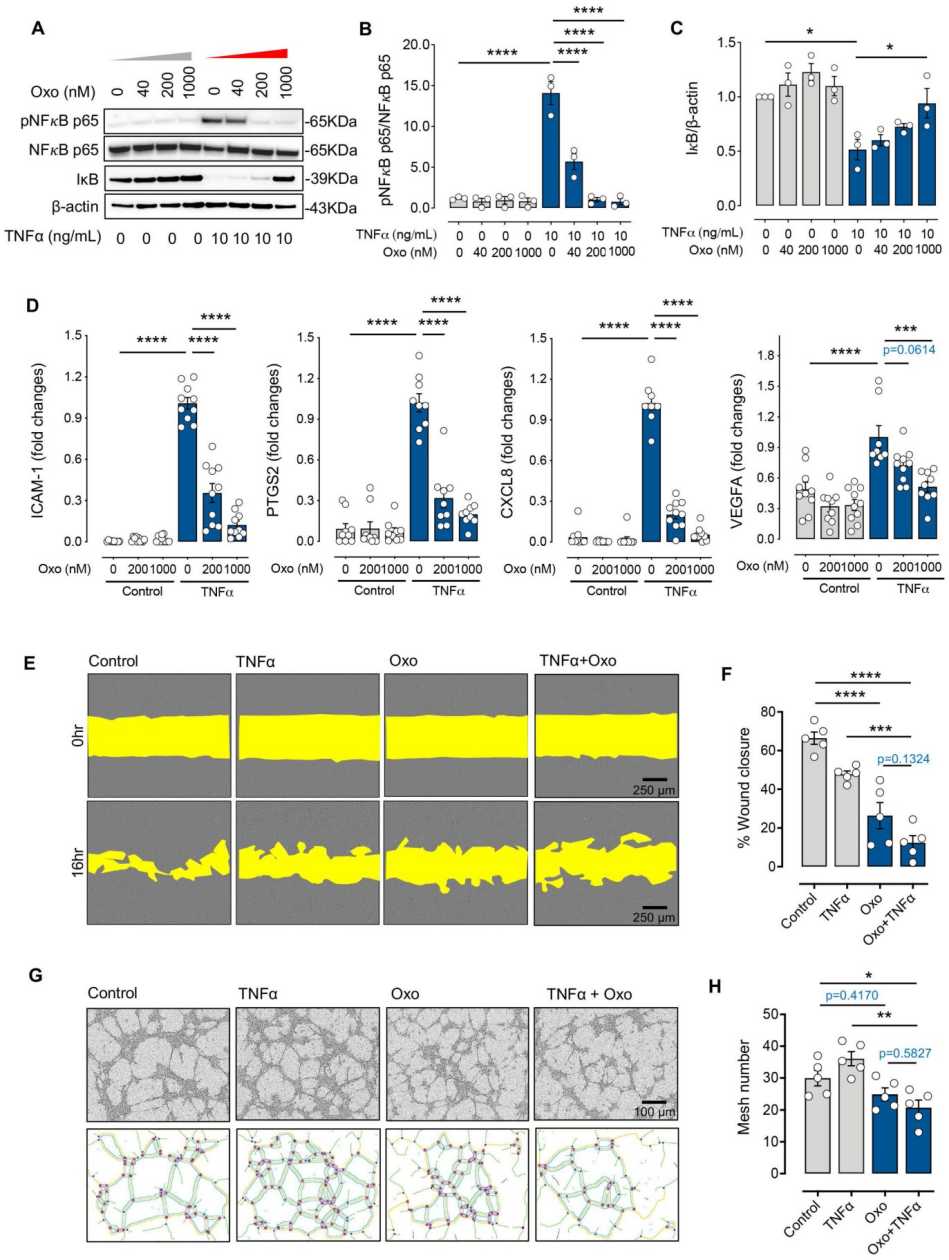
** 5Z-7-oxozeaenol suppresses inflammatory cytokine-mediated inflammatory signalling and angiogenic activities. (A)** Western blot characterization of the TNFα-induced phosphorylation of NFκB p65, and IκB proteins in TNFα (10 ng/mL for 10 minutes)-stimulated TIME cells. **(B and C)** Pharmaceutical inhibition of TAK1 reduced NFκB (NFκB p65 and IκB) signalling in TNFα-stimulated (10 ng/mL) TIME cells (n = 3 from three independent experiments) in a dose-dependent manner. **(D)** Gene expression of *ICAM1*, *PTGS2*, *CXCL8* and *VEGFA* was significantly suppressed by 200nM or 1000nM 5Z-7-oxozeaenol (Oxo) in TNFα-stimulated (10 ng/mL) TIME cells, assessed by qPCR (n = 9-10 from three independent experiments). **(E)** A wound healing assay was performed in TIME cells to evaluate the migration activity in the presence or absence of 5Z-7-oxozeaenol (Oxo) (1000 nM). Representative images of wound area were taken immediately after scraping and 24 hours after Oxo treatment. Scale bar: 250 µm. **(F)** Quantitative analysis was determined by healing distance normalised to the original wound distance (n = 5 from three independent experiments). **(G)** The tube formation assay was performed in the presence or absence of 5Z-7-oxozeaenol (Oxo) (1000 nM) in TIME cells. Representative images were taken 6 hours post tube formation. Scale bar: 100 µm. **(H)** Quantitative analysis was conducted by counting the lumen number (n = 5 from three independent experiments). Data is presented as means ± SEM. Statistical analysis was conducted by one-way ANOVA and Tukey's multiple comparison test. *P < 0.05, **P < 0.01, ***P < 0.001, ****P < 0.0001.

Previous studies indicate that TAK1 is activated in response to various proinflammatory stimuli such as TNFα and mediates downstream signalling related to both angiogenesis and inflammation through the nuclear factor kappa B (NFκB) pathway 16, 29. We assessed the effect of 5Z-7-oxozeaenol on the signalling pathway of NFκB and its downstream factors related to both angiogenesis and inflammation upon TNFα stimulation in human endothelial cells. TNFα stimulation clearly induced NFκB p65 phosphorylation in TIME cells, which was reduced with TAK1 inhibition by 5Z-7-oxozeaenol in a dose-dependent manner (**Figure 3A-B**). Inhibitor of NFκB (IκB) proteins are known to regulate NFκB activity. Its degradation and NFκB p65 phosphorylation are key events in NFκB signalling 30. TNFα stimulation resulted in IκB degradation in cells, while significant IκB expression was observed in cells treated with high doses of 5Z-7-oxozeaenol in the presence of TNFα (**Figure 3A and 3C**). Consistent with the inhibition of NFκB activity observed in 5Z-7-oxozeaenol-treated cells, 5Z-7-oxozeaenol effectively inhibited expression of TNFα-induced proinflammatory and proangiogenic genes, including *PTGS2*, *CXCL8* and *ICAM1* as well as *VEGF-A* (**Figure 3D**), which are known downstream genes of NFκB signalling 31, 32. Altogether, these results suggest that TAK1 plays a critical role in NFκB signalling under inflammatory insults. Interestingly, we also found that phosphorylation of JNK, p38, and ERK in MAPK signalling was decreased in cells treated with 5Z-7-oxozeaenol in a dose-dependent manner, suggesting that TAK1 inhibition also suppresses MAPK signalling (**Figure S5**), thus potentially inhibiting cell proliferation 33.

We further investigated the role of TAK1 in response to inflammatory insults in various endothelial functions related to angiogenesis through *in vitro* assays, including scratch migration and tube formation assay in TIME cells. Our results showed that TNFα treatment alone significantly reduced endothelial migration by 20% compared with controls, while cells treated with 5Z-7-oxozeaenol in the presence or absence of TNFα showed >50% reduction in cell migration (p<0.0001; **Figure 3E-F**). Furthermore, 5Z-7-oxozeaenol effectively reduced TNFα-induced tube formation activity in TIME cells (**Figure 3G-H**). Collectively, our data reveals that pharmacological inhibition of TAK1 significantly suppresses inflammation-mediated angiogenic signalling and reduces endothelial cell angiogenic activity.

### Formulation and characterization of gelatin nanoparticle-encapsulated 5Z-7-oxozeaenol

**Figure 4:**
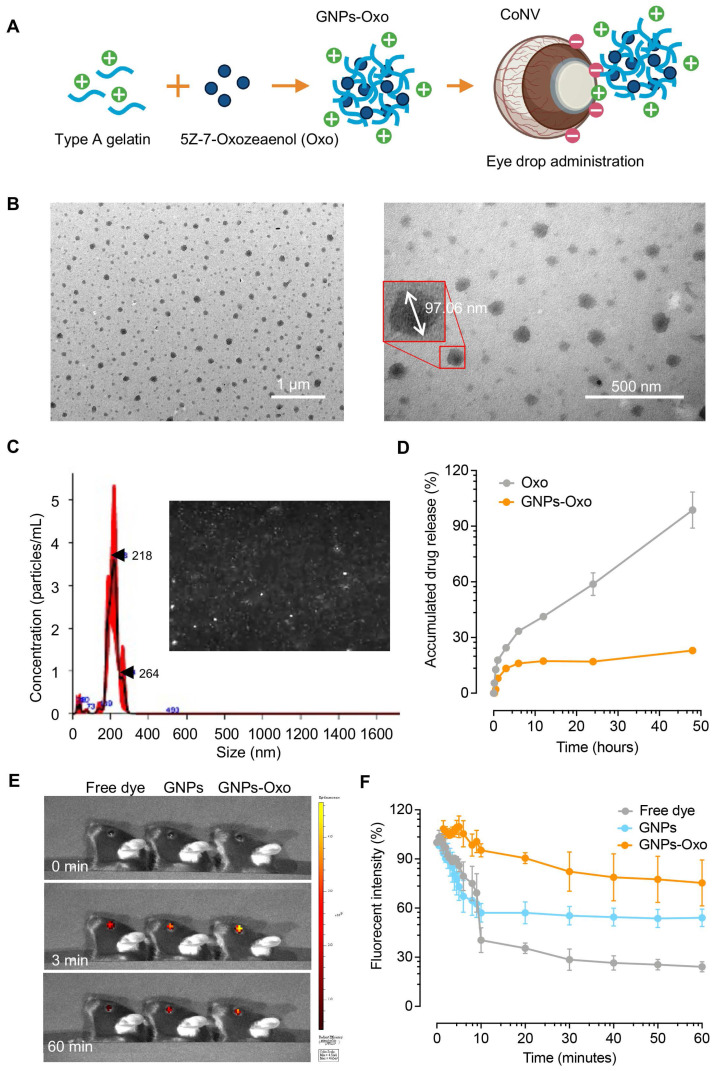
** Characterization of gelatin nanoparticle-encapsulated 5Z-7-oxozeaenol. (A)** A schematic diagram illustrates the formation of gelatin nanoparticle-encapsulated 5Z-7-oxozeaenol (Oxo). The positively charged gelatin nanoparticle (GNPs) was used to entrap 5Z-7-Oxozeaenol (Oxo) to form the nano-formulated GNPs-Oxo solution. Created with BioRender.com. **(B)** The polydispersity of GNPs-Oxo was scanned by transmission electron microscope. The size was ~97 nm in a well dispersed condition. Scale bar: 1 µm (left panel) and 500 nm (right panel). **(C)** The particle concentration and size of GNPs-Oxo particle was obtained by nanoparticle tracking analysis (NTA). Images represented the Brownian motion. **(D)** Drug release pattern of GNPs-Oxo compared to the free form of 5Z-7-Oxozeaenol (Oxo) at different time points (n = 3 from three independent experiments). **(E)** Ocular fluorescence from free dye (TAMRA), TAMRA-GNPs (GNPs), and TAMRA-GNPs-Oxo (GNPs-Oxo)-treated mice were photographed before eye drop instillment (0 min), and 3 minutes (3 min) or 60 minutes (60 min) post-instillment. **(F)** Ocular fluorescent intensity traced by IVIS at specific time point was quantified and represented as variation curve (n = 3 animals per treatment group). The percentage change of fluorescence intensity was individually compared with initial intensity. A piece-wise linear mixed-effects model was used to assess the rate of decay in fluorescence over time.

Conventional topical formulations, usually eye drops, comprise of 90% marketed ophthalmic pharmaceuticals, which are the least invasive approach for ocular drug administration 34, 35. Eye drop formulations are advantageous due to ease of administration, high acceptance by patients, and minimal systemic side effects 36. However, the major drawback is limited bioavailability (< 5%) due to multiple physical and biochemical barriers of the corneal surface. To overcome the limitations of current eyedrop formulations and prolong the effect of TAK1 inhibition in ocular surface, GNPs were used to formulate 5Z-7-oxozeaenol (GNPs-Oxo). A mouse model of chemical cauterization-induced CoNV was then employed to assess the antiangiogenic effect of topical application of GNPs-Oxo via eye drops (**Figure 4A**). We first manufactured GNPs-Oxo colloidal solution by a two-step desolvation method. Following the optimal parameter of GNPs from our previous study 37, the dynamic light scattering (DLS) results revealed that GNPs and GNPs-Oxo were prepared at the size of 137.3 ± 30.4 nm with zeta potential of 25.0 ± 6.4 mV and of 134.6 ± 28.3 nm and zeta potential of 25.5 ± 4.7 mV, respectively (**Table 1; Figure S6**). The poly dispersion index (PDI) in both groups was less than 0.2, which indicated that a monodisperse colloidal system was acquired (**Table 1; Figure S6**). Moreover, transmission electron microscopy (TEM) showed the synthesized GNPs-Oxo to be round and distinct particles with a spherical structure. The size of the particles was 80-130 nm in a well dispersed condition without aggregation (**Figure 4B**). The TEM images of GNPs showed a similar polydispersity to GNPs-Oxo (Data not shown). Nanoparticle tracking analysis (NTA), a method for visualizing and analyzing particles in liquid form, revealed that the particle distraction and size of GNPs-Oxo particles was similar to those assessed by DLS. The particle concentration of GNPs-Oxo in liquid was 2.25^11^ ± 2.87^10^ particles/mL with the size of 209 ± 40 nm (**Figure 4C**). A clearly slower release pattern of GNPs-Oxo was observed compared to the free form of 5Z-7-oxozeaenol at different time points (17.01% and 23% for GNPs-Oxo vs. 58.85% and 98.68% for the free form of 5Z-7-oxozeaenol at 24 and 48 hours, respectively; **Figure 4D**). The sustained-release of GNPs-Oxo can avoid rapid release of the drug, maintain effective concentration and prolong the retention of 5Z-7-oxozeaenol on ocular surface, thus enhancing ocular bioavailability in the eye. Notably, the GNPs-Oxo release was low (~20%) in this test, likely due to diffusion being the only driving force.

The distribution of nanoparticles conjugated with fluorescence dye (TAMRA) in the cell was examined using Image Press Pico imaging system. Cells displayed clearer staining in the cytoplasm and in the proximity of the nucleus 30 minutes post-treatment with GNPs-Oxo and increased staining intracellularly 2 hours post-treatment with GNPs-Oxo compared to cells treated with free dye alone, suggesting an enhanced cellular uptake of GNPs-Oxo (**Figure S7**). We speculate that easy uptake of GNPs might be due to higher lipophilicity of GNPs that facilitates nanoparticle fusion with cell membrane for endocytosis.

### Retention of gelatin nanoparticle-encapsulated 5Z-7-oxozeaenol on the mouse cornea

A fluorescence dye, TAMRA, was conjugated to GNPs and GNPs-Oxo particles to track the retention of formulated nanoparticles on the mouse cornea. To study ocular fluorescence from topical administration of the TAMRA solution (free dye), TAMRA-GNPs, or TAMRA-GNPs-Oxo, mouse corneas were imaged at multiple timepoints (**Figure 4E**). Quantification of fluorescence intensity revealed evident change among groups over time (**Figure 4F**). The average ocular fluorescence intensity of free dye dropped by ~60% 10 minutes after topical application compared with initial intensity. The average rate of resolution of free dye over the first 10 minutes was significantly faster than observed for TAMRA-GNPs-Oxo (*P* < 0.001) but similar to that of TAMRA-GNPs (*P* = 0.083). The average rate of resolution among the free dye and TAMRA-GNPs groups slowed significantly between 10 and 60 minutes compared to the first 10 minutes (*P* < 0.001 for each) but remained similar for TAMRA-GNPs-Oxo (*P* = 0.505) (**Table S2**). At 60 minutes after topical application, a significant reduction of particle retention in mice treated with free dye (average intensity [SD]: 24.2% [5.4]) was observed compared with those treated with TAMRA-GNPs (average intensity: 54.1% [9.2], *P* = 0.008) and TAMRA-GNPs-Oxo (average intensity: 75.3% (24.4), *P* = 0.024) (**Table S3**). As the outer-layer of tear film on the surface of the cornea is the lipid layer; we speculate that the GNP-Oxo readily interacts with the tear film to be endocytosed by corneal epithelial cells, resulting in the stronger fluorescence.

**Figure 5:**
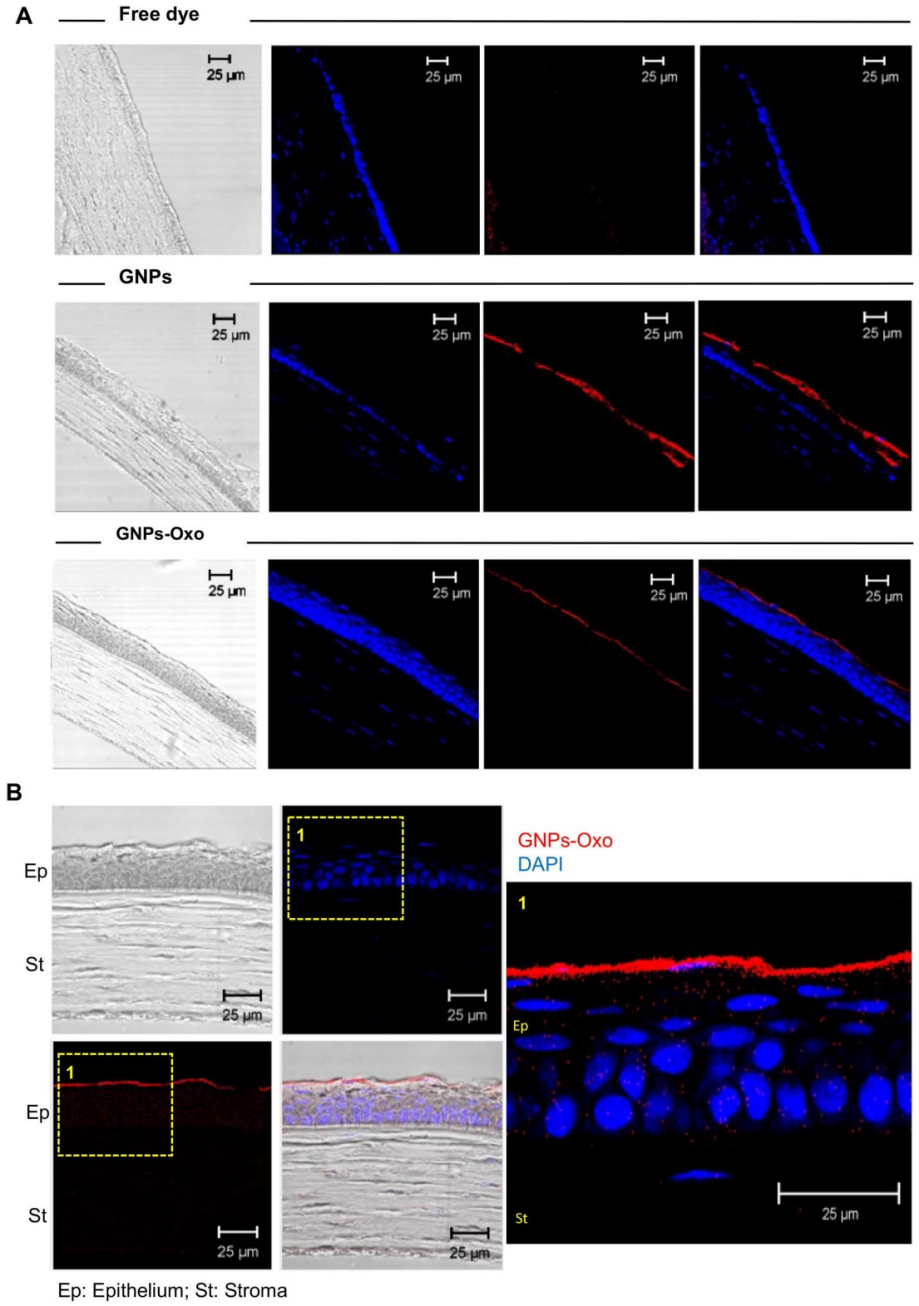
** Gelatin nanoparticle-encapsulated 5Z-7-oxozeaenol is retained in the mouse cornea. (A)** Corneal cryosections were examined by a confocal microscope 2 hours after topical application. Representative pictures of immunostaining of corneas from each of the three groups, including free dye (TAMRA), TAMRA-GNPs (GNPs), and TAMRA-GNPs-Oxo (GNPs-Oxo)-treated mice. Scale bar: 25 µm. **(B)** Nanoparticles were absorbed and located intracellularly in the cornea. The red fluorescence representing GNPs and GNPs-Oxo, but no free dye was observed in the upper layer of the cornea localized within the corneal epithelial layers. Scale bar: 25 µm. Ep: Epithelium; St: Stroma.

Subsequently, corneal cryosections were examined by a confocal microscope 2 hours after topical application. The red fluorescence representing TAMRA-conjugated GNPs and TAMRA-conjugated-GNPs-Oxo was observed in the upper layer of the cornea localized within the corneal epithelial layers (**Figure 5A**). However, there was no red fluorescence found in the cornea treated with free dye. The results provided direct evidence that GNPs and GNPs-Oxo were retained in the cornea. Nanoparticles were absorbed and located intracellularly with red fluorescence distribution only observed between nucleolus in corneal epithelial and stromal layers (**Figure 5B**). Overall, our data indicates that GNPs are able to sufficiently retain 5Z-7-oxozeaenol on the cornea. GNPs can also facilitate the drug to efficiently penetrate the deeper layers of the cornea.

### Topical application of gelatin nanoparticle-encapsulated 5Z-7-oxozeaenol suppresses the corneal neovascularization of a mouse model

**Figure 6:**
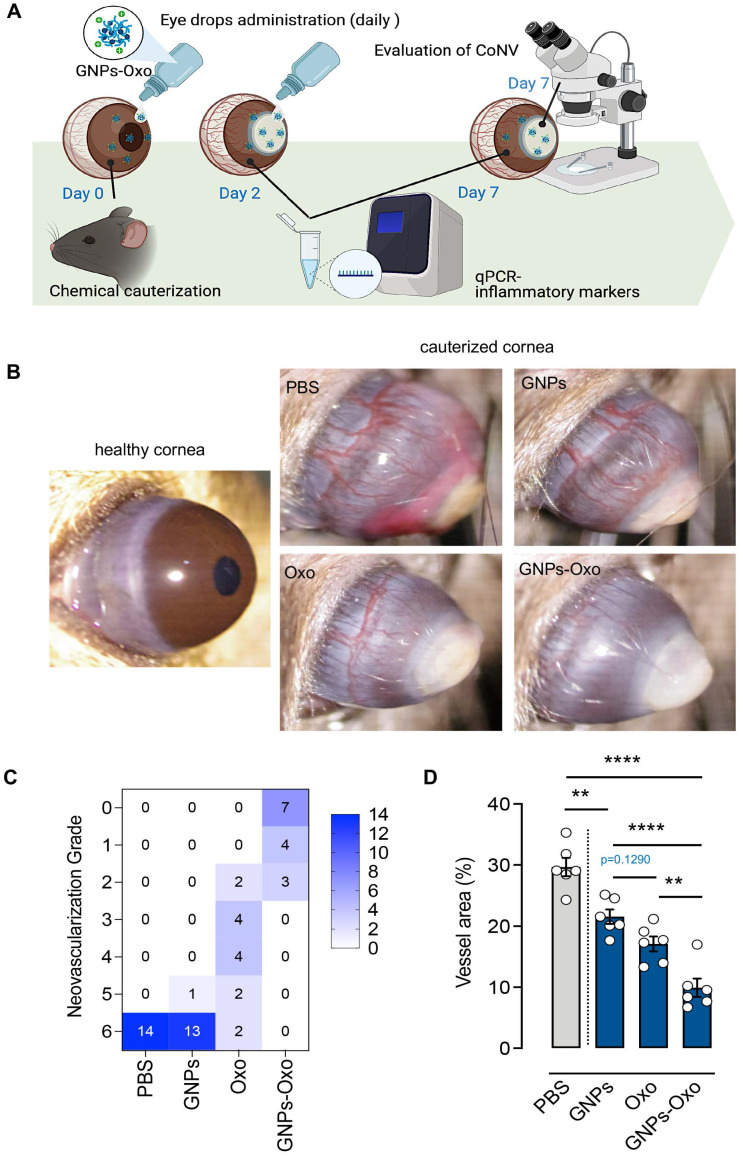
** Topical application of gelatin nanoparticle-encapsulated 5Z-7-oxozeaenol shows anti-angiogenesis effect in mouse model of chemical-cauterization-induced corneal neovascularization. (A)** A schematic diagram depicts the workflow of evaluation of GNPs-Oxo application in the animal model. C57BL/6J mouse eyes were chemically cauterized following a once daily topical application of PBS, GNPs, 5Z-7-oxozeaenol (Oxo), or GNPs-Oxo for 7 days. Created with BioRender.com. **(B)** Representative images were taken 7 days post treatment. **(C)** Quantification of neovascularization (NV) grade according to the extent of corneal NV (CoNV) was determined by the extent of vessel growth from limbus to burn edge (refer to methods for grading protocol) (n = 14 animals per treatment group from three independent experiments). **(D)** Quantification of vessel area (normalized to total cornea area) in the cornea (n = 6 corneas per treatment group). Statistical analysis was conducted by one-way ANOVA and Tukey's multiple comparison test; **P < 0.01, ****P < 0.0001.

Chemical cauterization in mice is one of the most commonly used methods to model human CoNV 38. To investigate the effect of GNPs-Oxo in CoNV, we compared the extent of neovascularization and the burn response in cauterized corneas treated with vehicle (PBS), GNPs, 5Z-7-oxozeaenol, or GNPs-Oxo. The topical administration of eye drops containing various subjects were given once a day for a consecutive 7 days to mice (**Figure 6A**). The radial growth of blood vessels from the corneal limbus towards the central burn area were observed 7 days after chemical cauterization (**Figure 6B**). Neovascularization grading showed that PBS and GNPs-treated mice had the highest score of 6 (PBS: 14/14 and GNPs: 13/14), suggesting that severe neovascularization had occurred in cornea. The score was significantly reduced in topical 5Z-7-oxozeaenol (score of 2-6) and GNPs-Oxo (score of 0-2) treated mice (**Figure 6C**). Quantitative analysis of CoNV revealed a significant reduction of neovascular area in mice treated with GNPs-Oxo compared with other groups (**Figure 6D**), suggesting that GNPs-Oxo provided a superior anti-angiogenic effect than the free form of 5Z-7-oxozeaenol.

Numerous studies have reported that various pro-inflammatory cytokines, such as IL-1β and TNFα, play critical roles in the pathogenesis of CoNV 38-41. Consistent with these findings, we found that gene expression of IL-1β and TNFα was significantly increased in mouse model of CoNV after 2 and 7 days of injury. A stronger expression of IL-1β and TNFα was mainly observed 7 days post-injury, suggesting relatively chronic inflammatory responses were developed in this model. Surprisingly, we found that IL-1β and TNFα expression was evidently increased in the mice treated with the free form of 5Z-7-oxozeaenol, while their expression was moderately reduced in those treated with GNPs-Oxo (**Figure S8A-B**). Moreover, quantitative analysis of corneal blisters, a severe corneal inflammatory response after chemical cauterization, showed no difference among groups (**Figure S8C**). Together, our results indicate that 5Z-7-oxozeaenol may not efficiently suppress inflammatory responses but is effective to inhibit pathological angiogenesis in the cornea.

## Discussion

The current therapeutic approaches for CoNV have limited efficacy and may result in a multitude of side effects. The long-term use of steroids, for example, may lead to the risk of superinfection, glaucoma or cataracts 7. Although the advent of anti-VEGF agents poised great hopes in managing CoNV, a few studies revealed that such treatment is only effective for newly formed vessels but not chronic neovascularization where mature vessels are covered by pericytes 3. TAK1 as a potential therapeutic target for pathologic angiogenesis has been widely studied as it closely engages in several important angiogenic activities, such as inflammatory response, hypoxia, and oxidative stress 42. Indeed, our previous study showed that TAK1 inhibition by 5Z-7-oxozeaenol effectively prevents retinal neovascularization 18, suggesting that the approach can be applied to other ocular neovascularization, such as CoNV. Conventional methods of drug delivery to cornea mainly rely on application of eye drops, an effective approach though with many limitations, such as poor ocular drug bioavailability, nasolacrimal duct drainage and poor penetration of cornea. We previously reported the gelatin-based nanoparticles as a vehicle to deliver drugs, which achieved significantly higher bioavailability 26. In the present study, we investigated the role of TAK1 in CoNV and revealed that anti-angiogenic effects of 5Z-7-oxozeaenol are mainly ascribed to inhibition of cell cycle and DNA replication. Moreover, gelatin nanoparticle-encapsulated 5Z-7-oxozeaenol proves superior compared with the free form of 5Z-7-oxozeaenol in terms of suppression of CoNV in a mouse model, due to extended retention of pharmaceutical ingredients.

TAK1 plays an indispensable role in regulating cell death and survival in many organs 16. TAK1 protects endothelial cells from death and maintains vascular integrity upon inflammatory insults, such as TNFα stimulation 19. Using endothelial-specific TAK1 knockout mice, a study demonstrated that TAK1 deficiency resulted in vascular destruction and rapid death due to endothelial apoptosis 19. Another study found that TAK1 deletion in endothelial cells led to increased cell death and vessel regression, while deletion of TNF signalling significantly protected endothelial cells from death in the TAK1-deleted embryos at embryonic day 10.5. In line with these studies, we also showed that TAK1 plays a protective role in endothelial cell apoptosis upon TNFα stimulation by using a selective inhibitor of TAK1, 5Z-7-oxozeaenol (data not shown). Moreover, 5Z-7-oxozeaenol effectively suppressed angiogenic activities upon TNFα stimulation, including cell migration and tube formation. Such effects are more likely due to inhibition of proliferation rather than the induced apoptosis. Indeed, 5Z-7-oxozeaenol directly inhibited endothelial cell proliferation and significantly reduced tube sprouting, as shown by cell viability and aortic ring assays, respectively. Previous studies have reported that TAK1 inhibition promotes cell cycle arrest and thus causes inhibition of cell proliferation in both retinal pigment epithelium cells and bone marrow-derived mesenchymal stem cells 43, 44. Our transcriptome analysis showed that TAK1 inhibition by 5Z-7-oxozeaenol resulted in down-regulation of several pathways related to cell growth and development, including cell cycle and DNA replication. Further pathway analysis revealed that TAK1 inhibition negatively affected all four phases of cell cycle and most molecules in DNA replication. Initiation of DNA replication, the core step in the S phase of cell cycle, is the converging point of growth regulatory pathways that control cell proliferation 45. Altogether, these results suggest that 5Z-7-oxozeaenol suppresses cell proliferation through inhibition of cell cycle and DNA proliferation, thus resulting in inhibition of angiogenesis.

TAK1 is known to control cell viability and inflammation through activating downstream signalling, such as NFκB pathway 16. Proinflammatory cytokines, such as TNFα, can activate NFκB pathway through the mediator, TAK1 46. TNFα binds to its receptor 1 (TNFR1) upon inflammatory stimuli, triggering TAK1 coupled with its binding protein TAB1-3 to phosphorylate inhibitor of NFκB (IκB) kinase at Ser^177^ and Ser^181^, which results in rapid proteasome-mediated degradation of IκB subunit. Consequently, NFκB (p50/p65) is activated and translocated to the nucleus to regulate gene expression 47. Our results revealed that pharmaceutical inhibition of TAK1 by 5Z-7-oxozeaenol in human endothelial cells prevented inflammatory stimuli from inducing sequential phosphorylation of upstream kinases in NFκB (p65) pathway and impeded expression of downstream genes associated with inflammation and angiogenesis, such as *ICAM1*, *PTGS2*, *CXCL8* and *VEGFA*. In addition, we also found that 5Z-7-oxozeaenol can effectively inhibit the JNK and p38 MAPK pathways (both mediated by MKK4 and MKK7) upon inflammatory stimuli. These data suggest an important role of TAK1 in crosstalk between inflammatory and angiogenic pathways, both of which are crucial in CoNV.

Physical barriers and the drainage system of cornea pose challenges for topical application of eye drops, largely due to its low bioavailability. To maintain minimum therapeutic dosage, frequent topical application of the eye drops is required. However, such administration potentially leads to poor patient compliance, and thereby poor efficacy of drugs. Pharmaceutical ingredients incorporated into nanoparticles have been studied for ocular applications extensively because it improves retention of the drug and increases permeation, therefore enhancing therapeutic efficacy by improving pharmacokinetics 48. The feasibility of nanomaterial modification provides the opportunity to adapt different pharmaceutical ingredients for various diseases 49. The cationic nanoparticles were found to prolong drug retention on the negatively-charged cornea, which enables more drug to enter the eye, allowing lower dosage and frequency of administration 50-52. Here, we demonstrated a clearly slower release pattern of GNPs-Oxo compared to the free form of 5Z-7-oxozeaenol over 48 hours by the dialysis method. In addition, we showed that GNPs prolonged 5Z-7-oxozeaenol residency on corneal surface* in vivo.* However, it is worth noting that the retention experiment was performed when animals were under anaesthesia, without rapid blinking and tear turnover. Therefore, the data must be interpreted cautiously as the role of the drug clearance might be underestimated after application of the eye drop. Further assessment of nanoparticle retention, such as ocular pharmacokinetics of GNPs-Oxo, should be performed to confirm prolonged drug retention by GNPs. We specifically applied the once-daily treatment instead of the normal 3-times daily treatment in animals to demonstrate that a once-daily treatment with the GNPs-Oxo was enough to provide an effective anti-angiogenesis effect in a mouse model of CoNV. The reduced treatment frequency suggests that the GNP-formulated drug is advantageous compared to its free form. Moreover, GNPs offer the advantages of improved biodegradability and biocompatibility, and drug-entrapment manufacturing is also easy 53, 54. Higher concentrations (250 µg/mL) of gelatin may also cause some cytotoxicity due to excessive and undegraded gelatin remnants accumulating in cells 37. Nevertheless, our previous study has demonstrated that GNPs did not cause significant changes in the central corneal thickness and intraocular pressure of rabbits 55, suggesting that GNPs are generally safe for corneal application.

Interestingly, we found that expression of inflammatory genes including IL-1β and TNFα in the mouse model of CoNV mildly rebounded in mice treated with 5Z-7-oxozeaenol alone compared with those treated with PBS, which can be ascribed to TAK1 inhibition in myeloid, neutrophil and macrophage cells 56, 57. In fact, neutrophils in the TAK1-deficient mice robustly produce proinflammatory cytokines upon activation, including IL-1β, TNFα, and IL-6, as well as reactive oxygen species 56. As neutrophil plays a critical role in the acute phase of inflammation-induced angiogenesis 58, 59, we suspect that TAK1 inhibition by 5Z-7-oxozeaenol may irritate neutrophils, thus causing a rebound of inflammatory cytokines in the CoNV mouse model. Nonetheless, GNPs-Oxo significantly diminished the rebound inflammatory response, which is likely due to less irritation of neutrophils 60, 61. Inflammation obviously comprises the pathologic process of CoNV, but inflammation-targeting therapies may not be ideal in treating CoNV. Although 5Z-7-oxozeaenol and GNPs-Oxo did not show anti-inflammatory effects as observed in other animal models, such as cerebral ischemia, arthritis, and retinal angiogenesis 18, 29, 62, we provide evidence that GNPs-Oxo is a useful approach to produce significant anti-angiogenic effect against chemical cauterization-induced CoNV *in vivo*. Moreover, to further manage the inflammatory response, combination therapy with steroids may be an attractive approach that can have an additive effect on the treatment of CoNV.

Our study illuminates avenues for further studies on other potential inhibitors of pathological angiogenesis, not limited to therapeutic targets still in experimental status but also those already in the market, which can be capsulated by GNPs. One example is tyrosine kinase inhibitors, FDA-approved anti-angiogenic drugs for cancer therapy, that enable inhibition of cell proliferation and migration 63. It would be interesting to see whether other GNPs-loaded anti-angiogenic agents can achieve a higher anti-angiogenic effect on CoNV than their free forms, similar to what we observed in this study. Of note, we chose to apply the relatively low dose (5 μg/mL) of 5Z-7-oxozeaenol to treat CoNV in a mouse model. This is because our result indicates that GNPs-Oxo eye drops at such low doses already provide a superior anti-angiogenic effect than the free form of 5Z-7-oxozeaenol. However, future studies will be required to investigate higher doses of the treatment, and ocular safety profiles must also be studied. Moreover, given the mouse model we used in this study was mainly induced by inflammation, it would be worthwhile to study efficacy of GNPs-Oxo in CoNV models with other induction methods, such as VEGF-induced or suture-induced model. Further studies should also investigate the efficacy of GNPs-Oxo beyond 7 days, as the duration of CoNV can last 14 days in this animal model.

## Conclusions

In summary, we demonstrate a novel therapeutic target, TAK1, for treating CoNV, and that gelatin nanoparticles are highly effective vehicles for drug delivery to the cornea. Our *in vitro* study reveals that pharmacologic inhibition of TAK1 by 5Z-7-oxozeaenol directly inhibits angiogenic activities of endothelial cells by inhibition of cell proliferation via cell cycle suppression and DNA replication. In addition, 5Z-7-oxozeaenol can inhibit both NFκB and NFκB-independent signalling, thereby suppressing angiogenesis. Furthermore, topical application of GNPs-Oxo significantly suppressed CoNV in a mouse model of CoNV, by disrupting endothelial cell proliferation, and exhibited anti-angiogenic effects rather than affecting cell inflammatory activities. Moreover, the positively charged GNPs prolong drug residency on cornea, suggesting a once-daily treatment to be adequate for providing significant anti-angiogenic effect.

## Supplementary Material

Supplementary figures and tables.

Supplementary databases 1.

Supplementary databases 2.

Supplementary databases 3.

Supplementary databases 4.

Sourced data and statistical analysis for non-supplementary figures.

## Figures and Tables

**Table 1:** Characterization of gelatin nanoparticle-encapsulated 5Z-7-oxozeaenol by dynamic light scattering (DLS) and high-performance liquid chromatography (HPLC)

	Size (nm)	Zeta (mV)	PDI	E.E. (%)	L.E. (%)
GNPs	137.3 ± 30.4	25.0 ± 6.4	0.158 ± 0.6	/	/
GNPs-Oxo	134.6 ± 28.3	25.5 ± 4.7	0.135 ± 0.2	54% ± 3%	1.25% ± 0.07%

Values represent mean ± standard division (n = 5); PDI: poly dispersive index; E.E: encapsulation efficiency; L.E.: loading efficiency; GNPs: gelatin nanoparticle; GNPs-Oxo: gelatin nanoparticle-5Z-7-oxozeaenol.

## Abbreviations

CoNV: Corneal neovascularization
DLS: Dynamic light scattering
GNP: Gelatin nanoparticle
GSEA: Gene set enrichment analysis
HPLC: High performance liquid chromatography
HUVEC: Human umbilical vein endothelial cell
IL-1: Interleukin-1
IκB: Inhibitor of NFκB
NFκB: Nuclear factor kappa B
NTA: Nanoparticle tracking analysis
Oxo: 5Z-7-oxozeaenol
PDI: Particle polydispersity index
PDT: Photodynamic therapy
qPCR: Quantitative polymerase chain reaction
RNASeq: RNA sequencing
TAK1: Transforming growth factor-β-activated kinase 1
TAMRA: 5-Carboxytetramethylrhodamine succinimidyl ester
TEM: Transmission electron microscopy
TGFβ: Transforming growth factor β
TIME: Telomerase-immortalized human microvascular endothelium
TLR: Toll-like receptor
TNFα: Tumor necrosis factor α
TNFR1: TNFα binds to its receptor 1
VEGF: Vascular endothelium growth factor
