# Supplementary material for: Topical application of TAK1 inhibitor encapsulated by gelatin particle alleviates corneal neovascularization
Source: Theranostics. 2022 Jan 1;12(2):657–74. doi: 10.7150/thno.65098 (PMC8692906; doi:10.7150/thno.65098)
Supplement: Supplementary file 1 — Supplementary figures and tables. [file thnov12p0657s1.pdf]

Supplementary tables and figures

Topical application of TAK1 inhibitor encapsulated by gelatin particle alleviates corneal neovascularization

Jiang-Hui Wang[1†], Ching-Li Tseng[2†], Fan-Li Lin[3,4], Jinying Chen[5], Erh-Hsuan Hsieh[2], Suraj Lama[3], Yu-Fan Chuang[3,4], Satheesh Kumar[3], Linxin Zhu[3], Myra B. McGuinness[1,6], Jessika Hernandez[3], Leilei Tu[5], Peng-Yuan Wang[4], Guei-Sheung Liu[1,2,3,7,8*]

[1]Centre for Eye Research Australia, Royal Victorian Eye and Ear Hospital, East Melbourne, Australia
[2]Graduate Institute of Biomedical Materials and Tissue Engineering, College of Biomedical Engineering, Taipei Medical University, Taipei, Taiwan
[3]Menzies Institute for Medical Research, University of Tasmania, Hobart, Australia
[4]Shenzhen Key Laboratory of Biomimetic Materials and Cellular Immunomodulation, Shenzhen Institute of Advanced Technology, Chinese Academy of Sciences, Shenzhen, China
[5]Department of Ophthalmology, the First Affiliated Hospital of Jinan University, Guangzhou, China
[6]Centre for Epidemiology and Biostatistics, Melbourne School of Population and Global Health, University of Melbourne, Melbourne, Australia
[7]Ophthalmology, Department of Surgery, University of Melbourne, East Melbourne, Australia
[8]Aier Eye Institute, Changsha, Hunan, China

†JHW and CLT contributed equally to this work

*Correspondence and requests for materials should be addressed to
Dr Guei-Sheung Liu (rickliu0817@gmail.com). Menzies Institute for Medical Research, University of Tasmania. Address: 17 Liverpool Street, Hobart, TAS 7000, Australia. Tel: +61362264250.

Supplementary tables

Table S1 Key Resources table

REAGENT or RESOURCE	SOURCE	IDENTIFIER
**Antibodies**		
IκBα (L35A5) mouse mAb	Cell Signaling Technology	4814
Phospho-NFκB p65 (Ser536) (93H1) rabbit mAb	Cell Signaling Technology	3033
NFκB p65 (C22B4) rabbit mAb	Cell Signaling Technology	4764
Phospho-p44/42 MAPK (Erk1/2) (Thr202/Tyr204) rabbit pAb	Cell Signaling Technology	9101
p44/42 MAPK (Erk1/2) (137F5) rabbit mAb	Cell Signaling Technology	4695
Phospho-SAPK/JNK (Thr183/Tyr185) (G9) mouse mAb	Cell Signaling Technology	9255
SAPK/JNK rabbit pAb	Cell Signaling Technology	9252
Phospho-p38 MAPK (Thr180/Tyr182) rabbit pAb	Cell Signaling Technology	9211
p38 MAPK (D13E1) XP® rabbit mAb	Cell Signaling Technology	8690
Actin (clone C4) mouse mAb	Merck Millipore	MAB1501
Goat anti-mouse IgG HRP-conjugated secondary Ab	Life Technologies Australia	31430
Goat anti-rabbit IgG HRP-conjugated secondary Ab	Life Technologies Australia	656120
**Chemicals**		
5Z-7-Oxozeaenol	Tocris Bioscience	3604/1
Recombinant Human TNFα protein	Gibco™	PHC3015
Recombinant Human VEGF165 Protein	R&D Systems	293-VE-010
Type A gelatin (bloom 175)	Sigma-Aldrich	G2625
DMSO	JT Baker	9224-01
Glutaraldehyde	Sigma-Aldrich	G6257
Acetone	Seedchem Company	AC0061
Uranium acetate	Thermo Fisher Scientific	541-09-3
Acetonitrile	Aencore Chemical	AE0627
5-Carboxytetramethylrhodamine, succinimidyl ester (5-TAMRA)	Thermo Fisher Scientific	C2211
Zoletil 50®	Virbac	n/a
Rompun®	Bayer	n/a
Alcaine®	Alcon	n/a
Silver Nitrate Applicators 6 inch	Grafco	1590
DAPI	Generon	40043
**Critical Commercial Assays**		
CYQUANT® NF cell proliferation assay kit	Life Technologies	C35006

	Australia	
Quick-RNA MiniPrep kit	Zymo Research	R1055
High-capacity cDNA reverse transcription Kit	Life Technologies Australia	4368814
TaqMan™ Fast Advanced Master Mix	Life Technologies Australia	4444553
Pierce™ BCA assay kit	Life Technologies Australia	23227
Amersham ECL Prime Western Blotting Detection Kit	GE Healthcare	RPN2232
MycoAlert™ Mycoplasma Detection Kit	Lonza	LT07
**Experimental Models: Cell Lines**		
Telomerase-immortalized human microvascular endothelium cell (TIME)*<br>*Cell was derived from a primary culture of neonatal foreskin microvascular endothelial cells of the dermis.	ATCC	CRL-4025™
Human Umbilical Vein Endothelial Cell (HUVEC)*<br>*Cells was derived from the endothelium of veins from the umbilical cord.	Life Technologies Australia	C0035C
**Experimental Models: Organisms/Strains**		
C57BL/6 mouse	Taipei Medical University	LAC-2015-0328 and LAC-2019-063
**qPCR Probes**		
Human VEGFA TaqMan probe	Applied Biosystems	Hs00900055_m1
Human ICAM1 TaqMan probe	Applied Biosystems	Hs00164932_m1
Human PTGS2 TaqMan probe	Applied Biosystems	Hs00153133_m1
Human CXCL8 TaqMan probe	Applied Biosystems	Hs00174103_m1
Human GAPDH TaqMan probe	Applied Biosystems	Hs99999905_m1
Mouse TNFa TaqMan probe	Applied Biosystems	Mm00443258_m1
Mouse IL1b TaqMan probe	Applied Biosystems	Mm00434228_m1
Mouse actin-b TaqMan probe	Applied Biosystems	Mm00607939_s1
**Software and Algorithms**		
Image Pro Plus	Media Cybernetics	n/a
ImageJ (v1.48)	Schneider et al., 2012 https://imagej.nih.gov/ij	n/a
Adobe Photoshop	Adobe.<br>Connor et al., 2009	n/a
GraphPad Prism 7	GraphPad Software	n/a
R (v4.0.2)	Bioconductor	n/a
Trimgalore (v0.4.4)	Babraham Institute	n/a
STAR (v2.5.3)	Alexander Dobin	n/a
FeatureCounts (subread 1.6.4)	Liao Y, Smyth GK and Shi W., 2014	n/a
**Other**		
Matrigel™ Basement Membrane Matrix	Corning	356234
Pierce RIPA buffer	Life Technologies	89900

	Australia	
Protease inhibitor cocktail	Roche Diagnostics	11697498001
Bolt™ 4-12% Bis-Tris Plus Gel	Invitrogen	NW04120BOX
Immuno-Blot® PVDF Membrane	Bio-Rad Laboratories	1620177
EBM™ Plus Basal Medium supplied with EGM™ Plus SingleQuots™ supplements	Lonza	CC-5035
Modified Davidson's fluid	CIS-Bio, Taiwan	D476
10 % neutral buffered formalin solution	CIS-Bio, Taiwan	D468
FSC22 Frozen section media	Leica	3801480
Fluoromount™ aqueous mounting medium	Sigma-Aldrich	F-4680
Incucyte® 96-well WoundMaker Tool	Incucyte	4563
TrypLE Express Enzyme (1X)	Life Technologies Australia	12605028
Spectra/Por, Float-A-Lyzer G2 Dialysis Device (MWCO 20 kDa)	Sigma-Aldrich	Z726710-12EA

Table S2 Estimated rate of change in fluorescent intensity from a piecewise linear mixed-effects model

	Dye	Average rate of change		Difference to free dye			Difference to first 10 mins		
		%/minute	(95% CI)	%/minute	(95% CI)	p-value*	%/minute	(95% CI)	p-value*
0-10 mins	Free dye	-5.4	(-6.2,-4.7)						
	TAMRA-GNPs	-4.5	(-5.2,-3.7)	0.9	(-0.1,2.0)	0.083			
	TAMRA-GNPs-Oxo	-0.8	(-1.6,-0.1)	4.6	(3.5,5.7)	<0.001			
>10-60 mins	Free dye	-0.7	(-1.1,-0.4)				4.7	(3.9,5.5)	<0.001
	TAMRA-GNPs	0.0	(-0.3,0.3)	0.7	(0.3,1.2)	0.002	4.5	(3.7,5.3)	<0.001
	TAMRA-GNPs-Oxo	-0.5	(-0.9,-0.2)	0.2	(-0.2,0.7)	0.380	0.3	(-0.5,1.1)	0.505

* Derived from a piecewise linear mixed-effects model with random intercepts and slopes

Table S3 Observed values at specific time points from Figure 4F

Time	Dye	Fluorescent intensity (%)	Difference free dye (%)	
		Average (SD)	Average	p-value*
10 mins	Free dye	40.4 (13.1)		
	TAMRA-GNPs	57.1 (9.8)	16.7 (9.5)	0.153
	TAMRA-GNPs-Oxo	95.3 (6.9)	54.9 (8.5)	0.003
60 mins	Free dye	24.2 (5.4)		
	TAMRA-GNPs	54.1 (9.2)	29.9 (6.1)	0.008
	TAMRA-GNPs-Oxo	75.3 (24.4)	51.2 (14.4)	0.024

* Derived from two sample t-tests

Supplementary figures

Figure S1

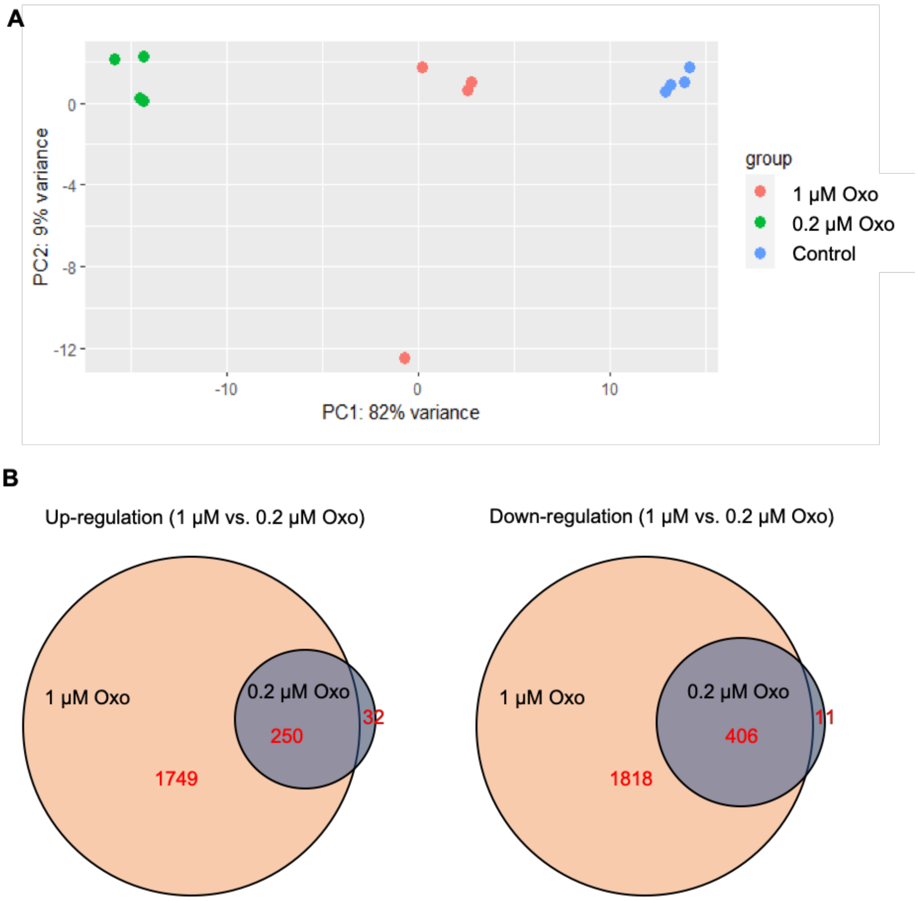

Figure S1 (A) Principal component analysis of RNA-seq data of control, 0.2 µM and 1 µM 5Z-7-oxozeaenol treated human telomerase-immortalized human microvascular endothelial (TIME) cells. Each dot represents an experimental replicate. **(B)** Venn diagram shows the overlapped dysregulated genes between dysregulated genes identified in 0.2 µM and 1 µM 5Z-7-oxozeaenol treated TIME cells.

Figure S2

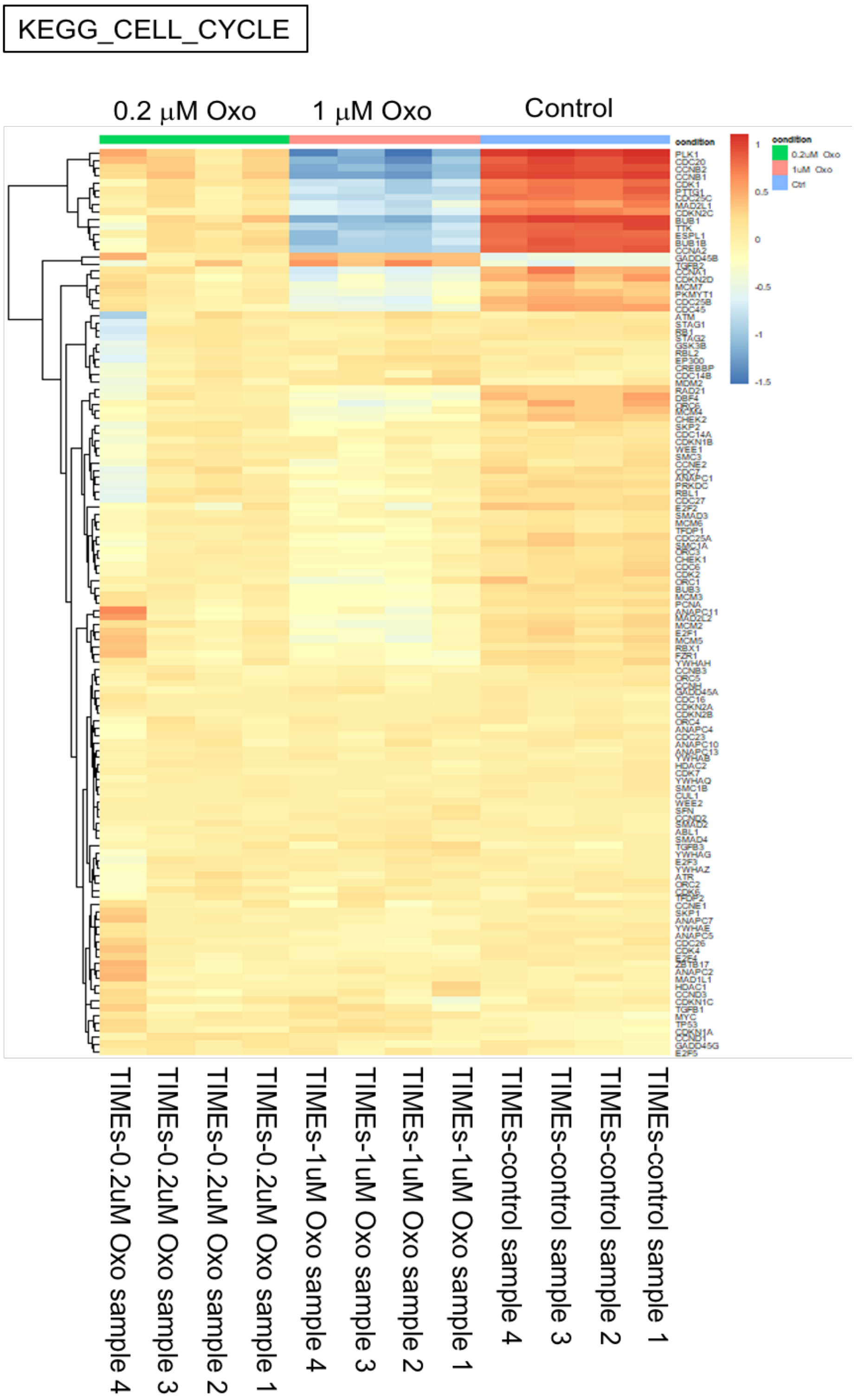

Figure S2 Heat map generated from unsupervised clustering of genes from gene set of 'cell cycle' (Reactome Pathway Database) of 0.2 µM and 1 µM 5Z-7-oxozeaenol treated TIME cells and normal controls.

Figure S3

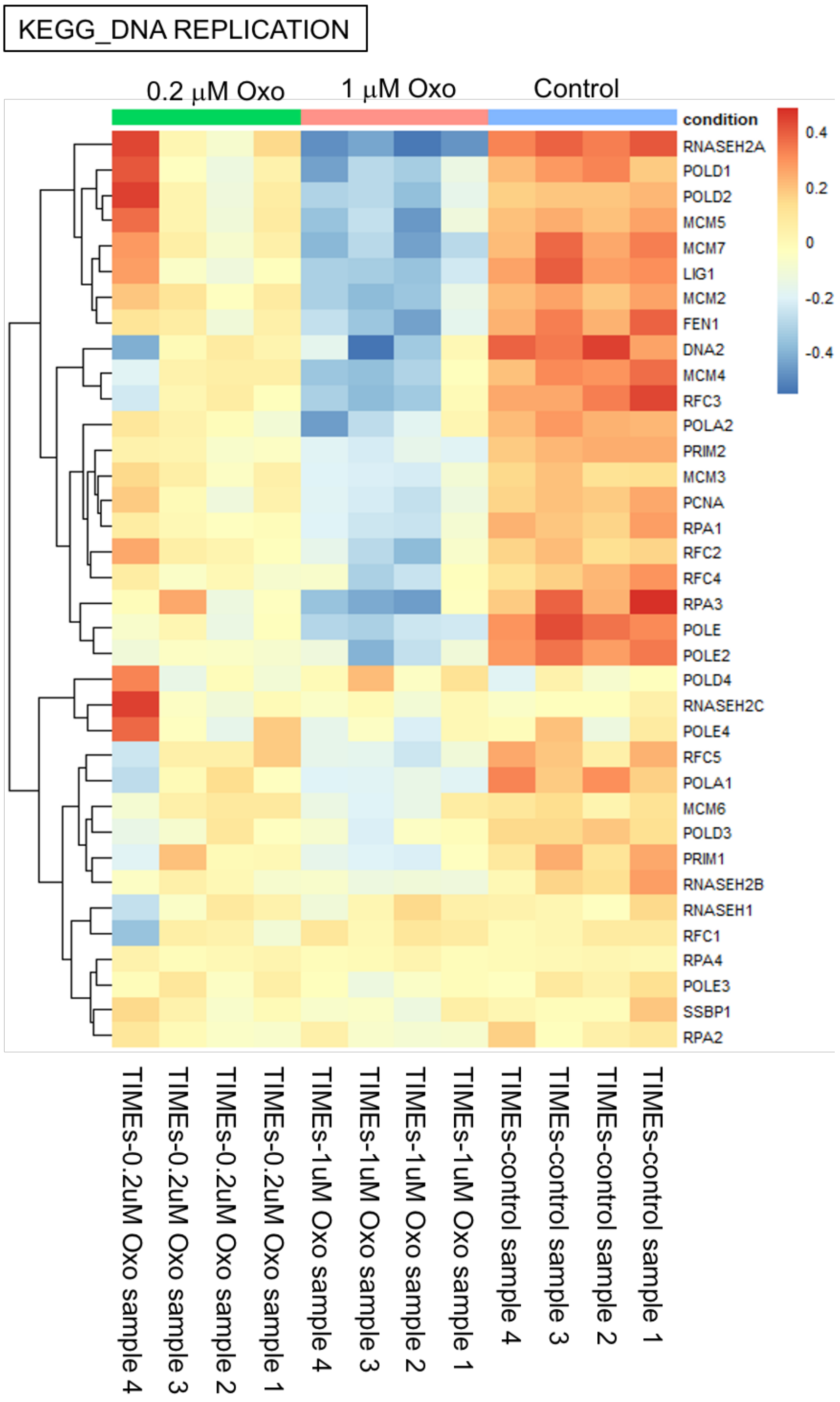

Figure S3 Heat map generated from unsupervised clustering of genes from gene set of 'DNA Replication' (Reactome Pathway Database) of 0.2 μM and 1 μM 5Z-7-oxozeaenol treated TIME cells and normal controls.

Figure S4

A

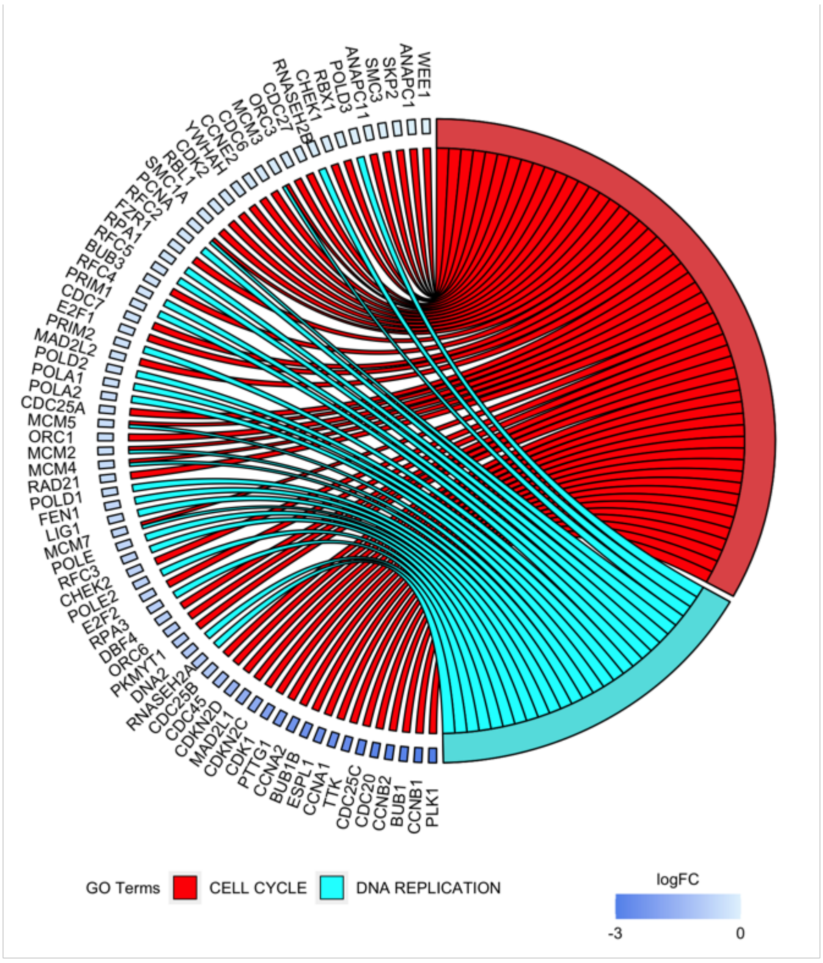

B

KEGG_ Cell Cycle: Control vs. 1 μM Oxo

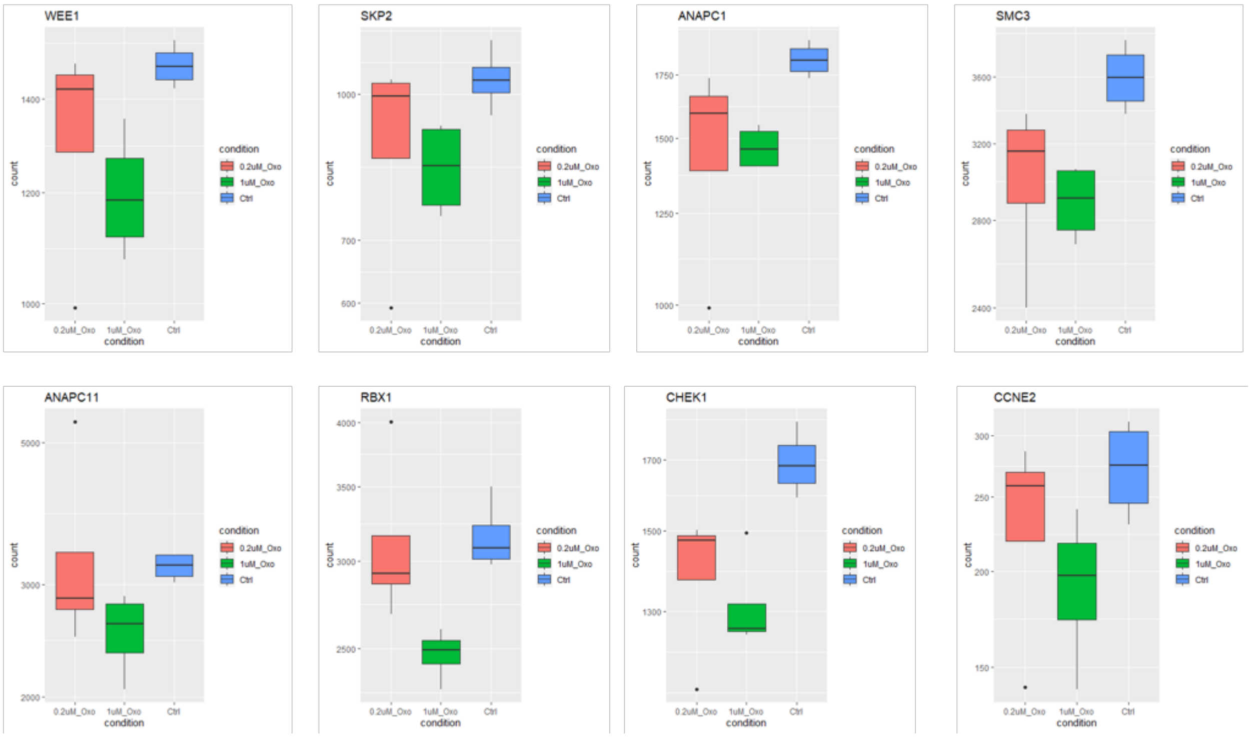

KEGG_ Cell Cycle: Control vs. 1 μM Oxo

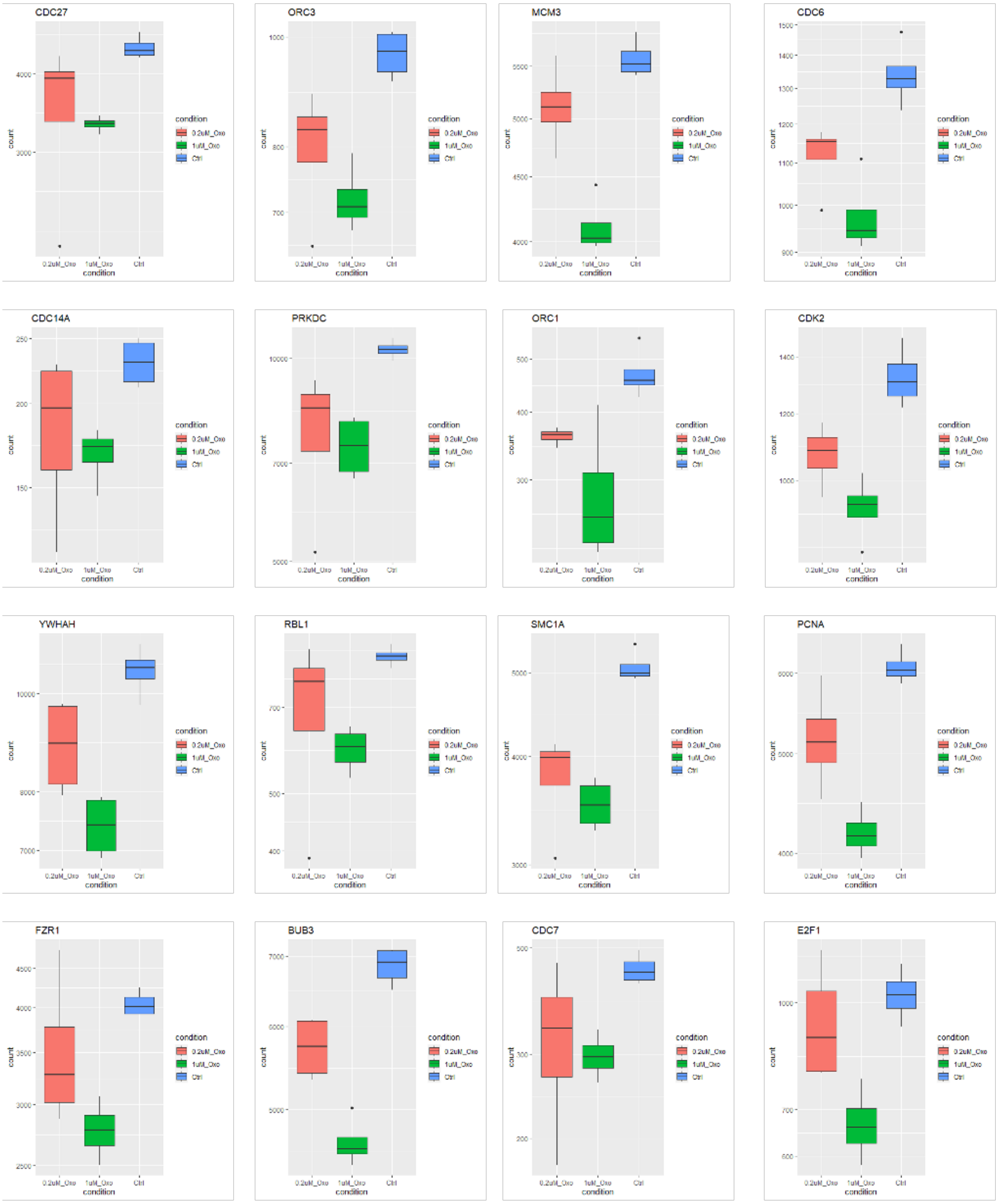

KEGG_ Cell Cycle: Control vs. 1 µM Oxo

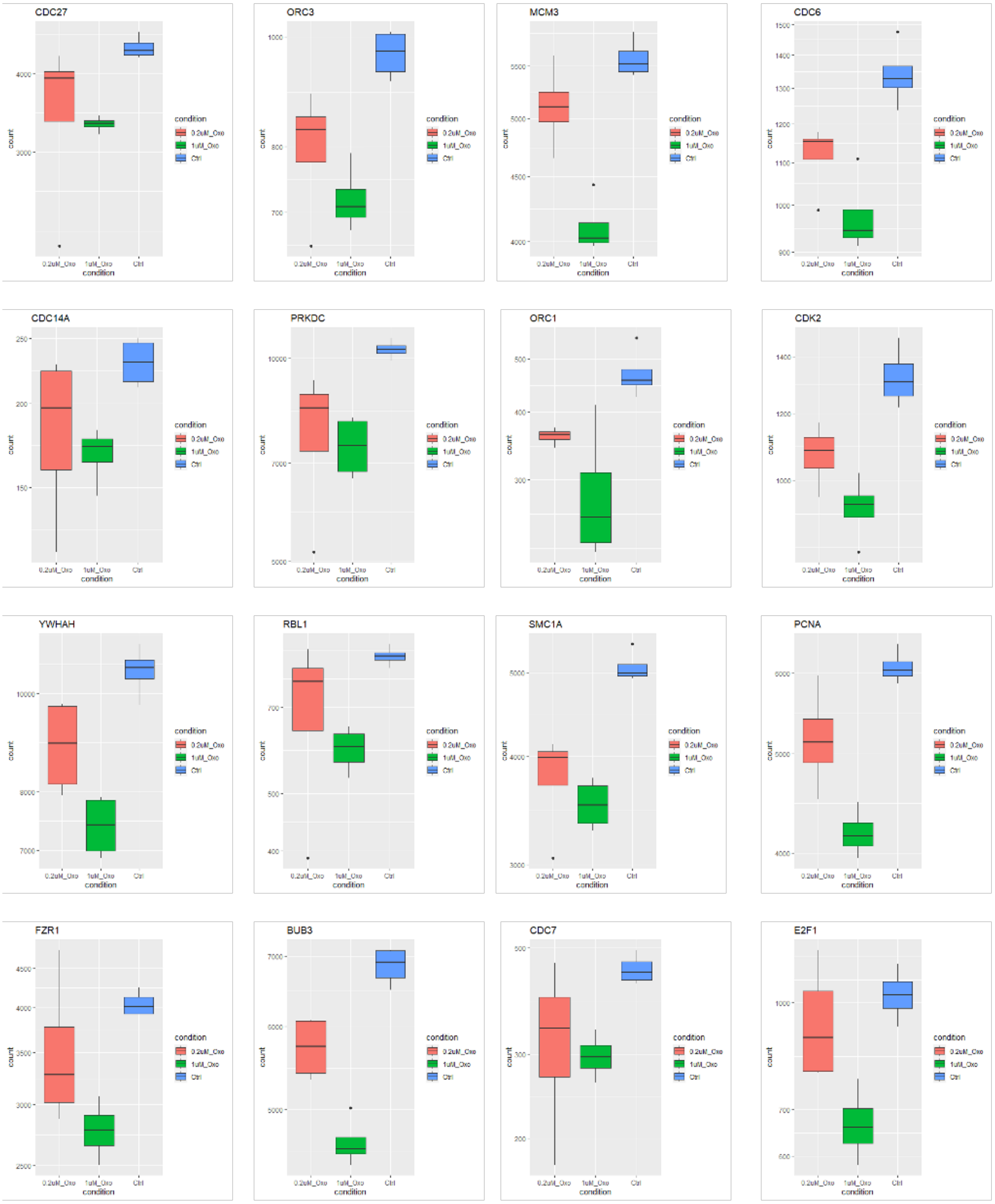

KEGG_ Cell Cycle: Control vs. 1 µM Oxo

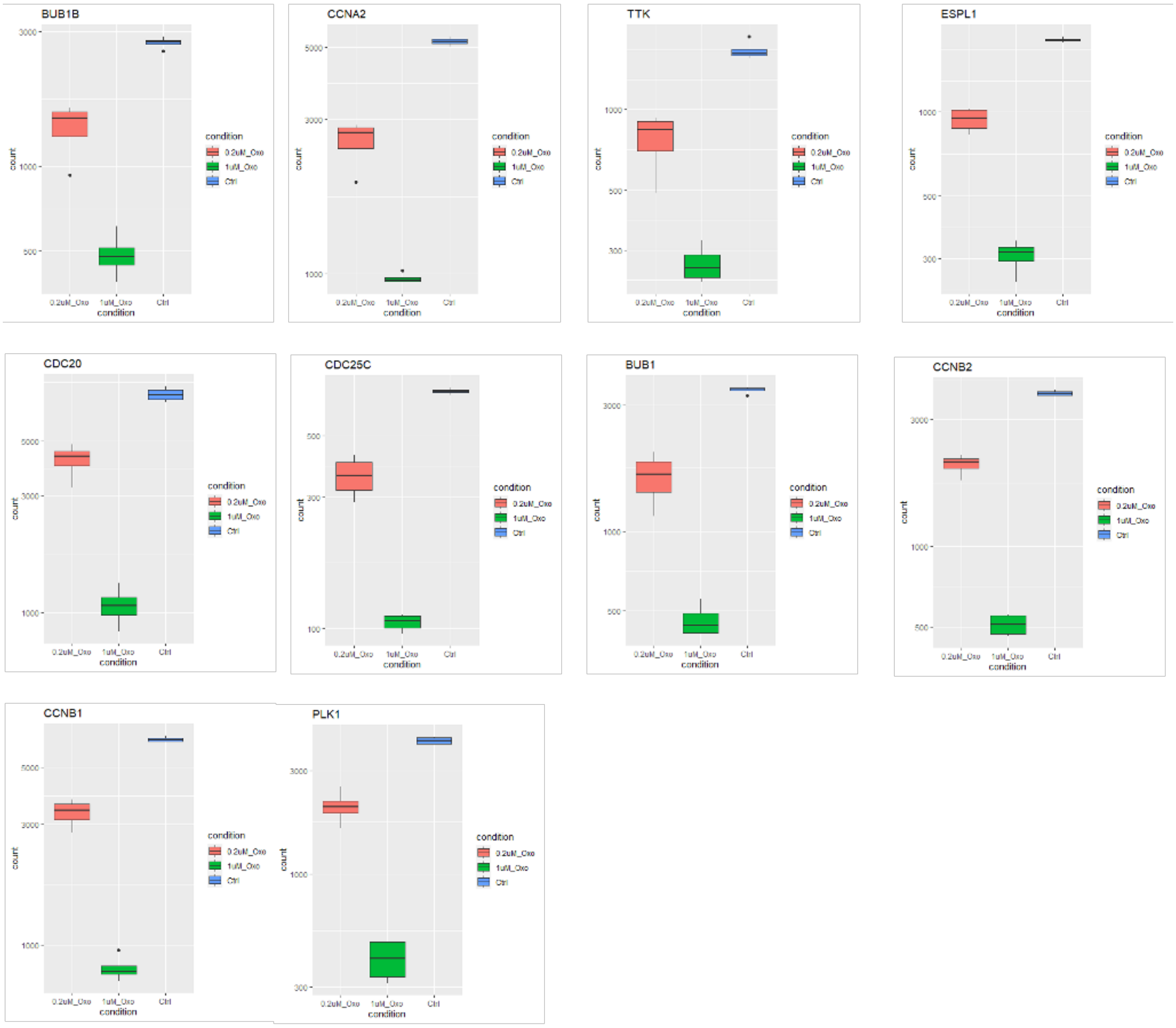

Figure S4 (A) Circular plot of KEGG pathway (cell cycle and DNA replication) enrichment analysis. **(B)** Bar charts show the normalized read count of each gene in the gene set of ‘Cell Cycle’ identified by GSEA study in TIME cells treated with 1 µM 5Z-7-oxozeaenol. **(C)** Bar charts show the normalized read count of each gene in the gene set of ‘DNA Replication’ identified by GSEA study in TIME cells treated with 1 µM 5Z-7-oxozeaenol.

Figure S5

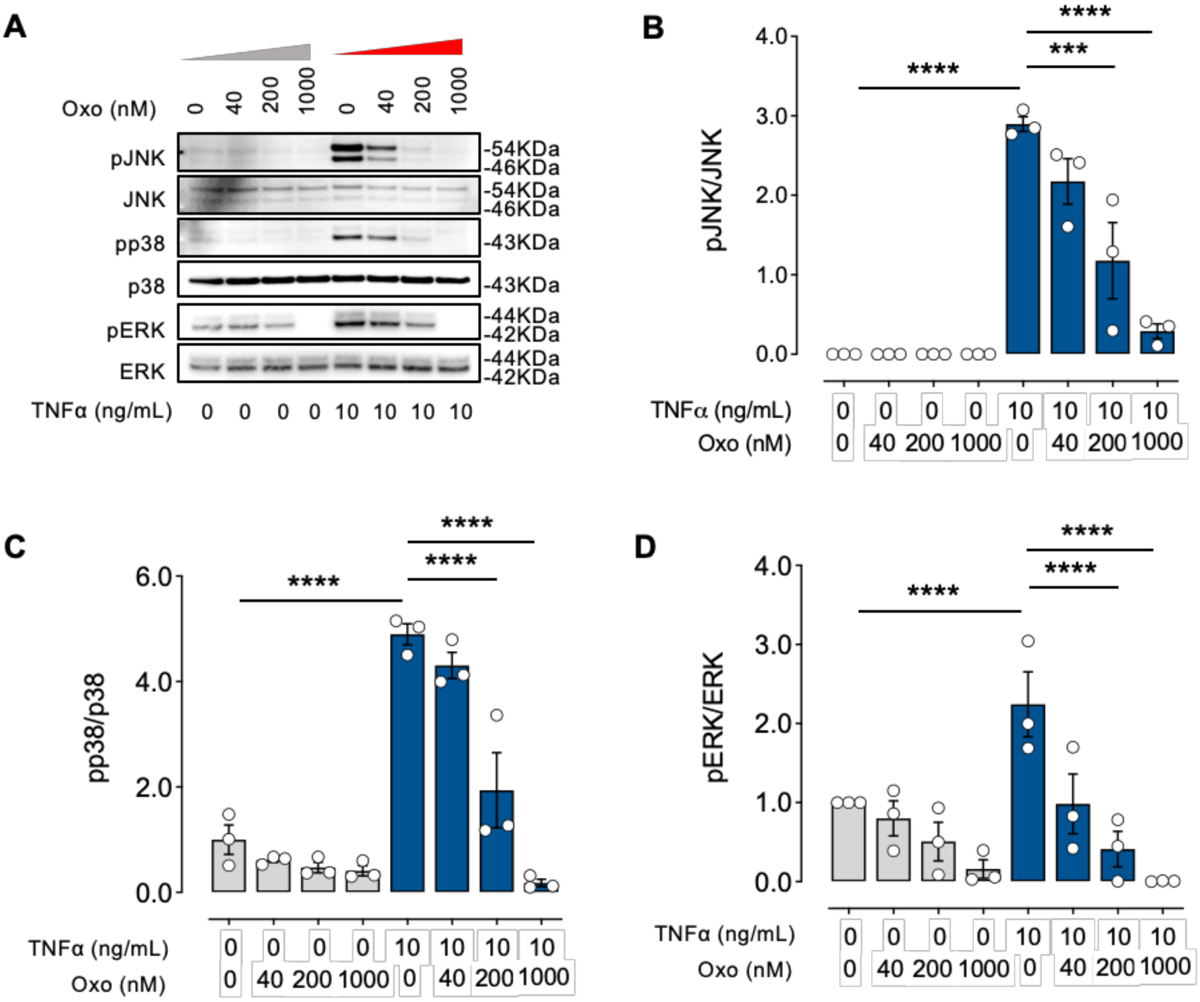

Figure S5 5Z-7-oxozeaenol suppresses inflammatory cytokine-mediated MAPK signaling. (A) Western blot characterization of the TNFα-induced phosphorylation of JNK, p38, and ERK proteins in TIME cell stimulated by TNFα (10 ng/ml) for 10 minutes. **(B-D)** Phosphorylation of JNK, p38, and ERK proteins in MAPK signaling was decreased in cells treated with 5Z-7-oxozeaenol in a dose-dependent manner (n = 3 from three experiments). Statistical analysis was conducted by one-way ANOVA and Tukey's multiple comparison test. ****P* < 0.001, *****P* < 0.0001.

Figure S6

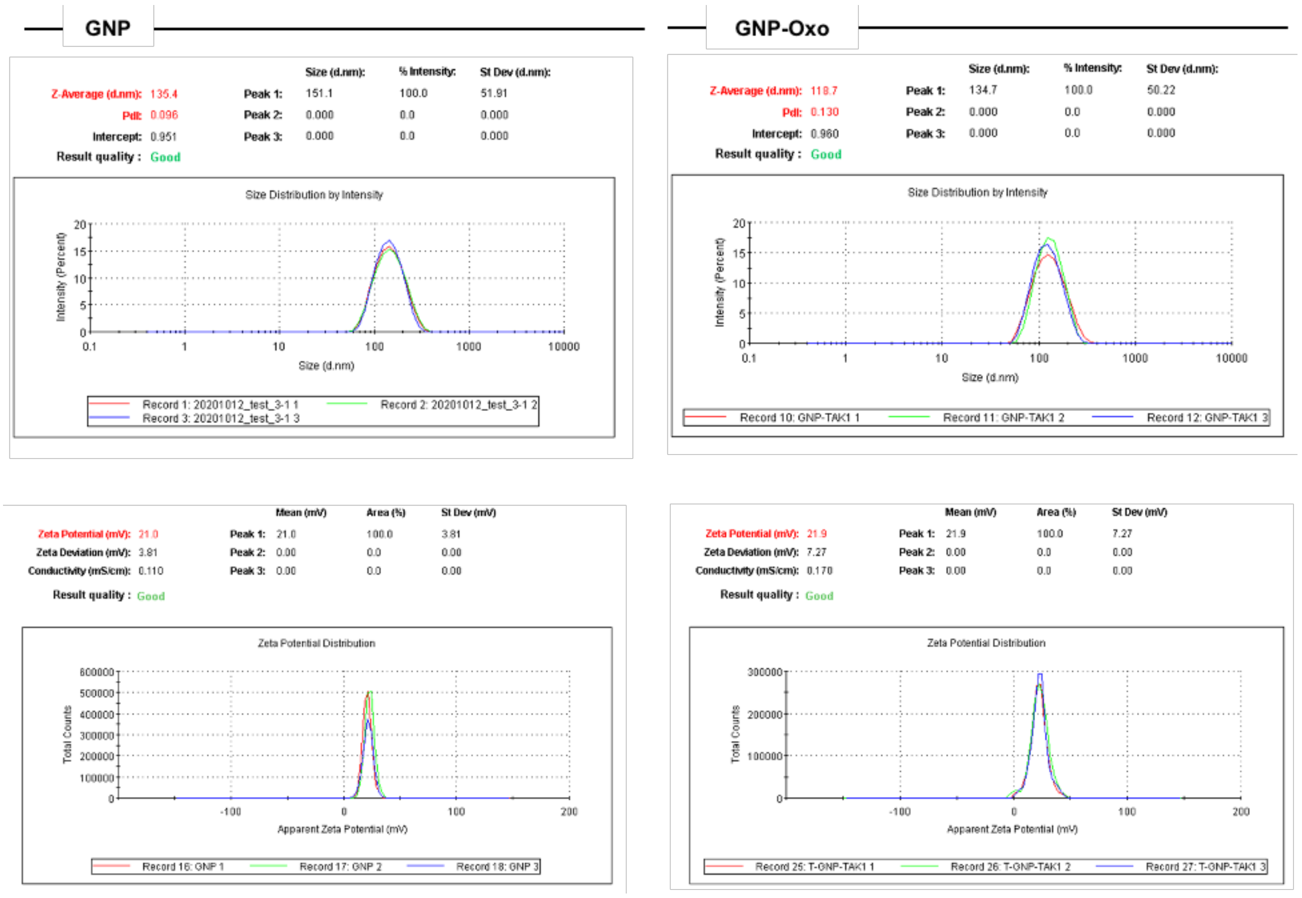

Figure S6 Characterization of gelatin nanoparticle-encapsulated 5Z-7-oxozeaenol. Representative images from dynamic light scattering (DLS) analysis revealed the particle polydispersity index (PDI), size, and zeta potential of GNP and GNP-Oxo particles. Each sample was tested in triplicates, shown as red, green, and blue lines.

Figure S7

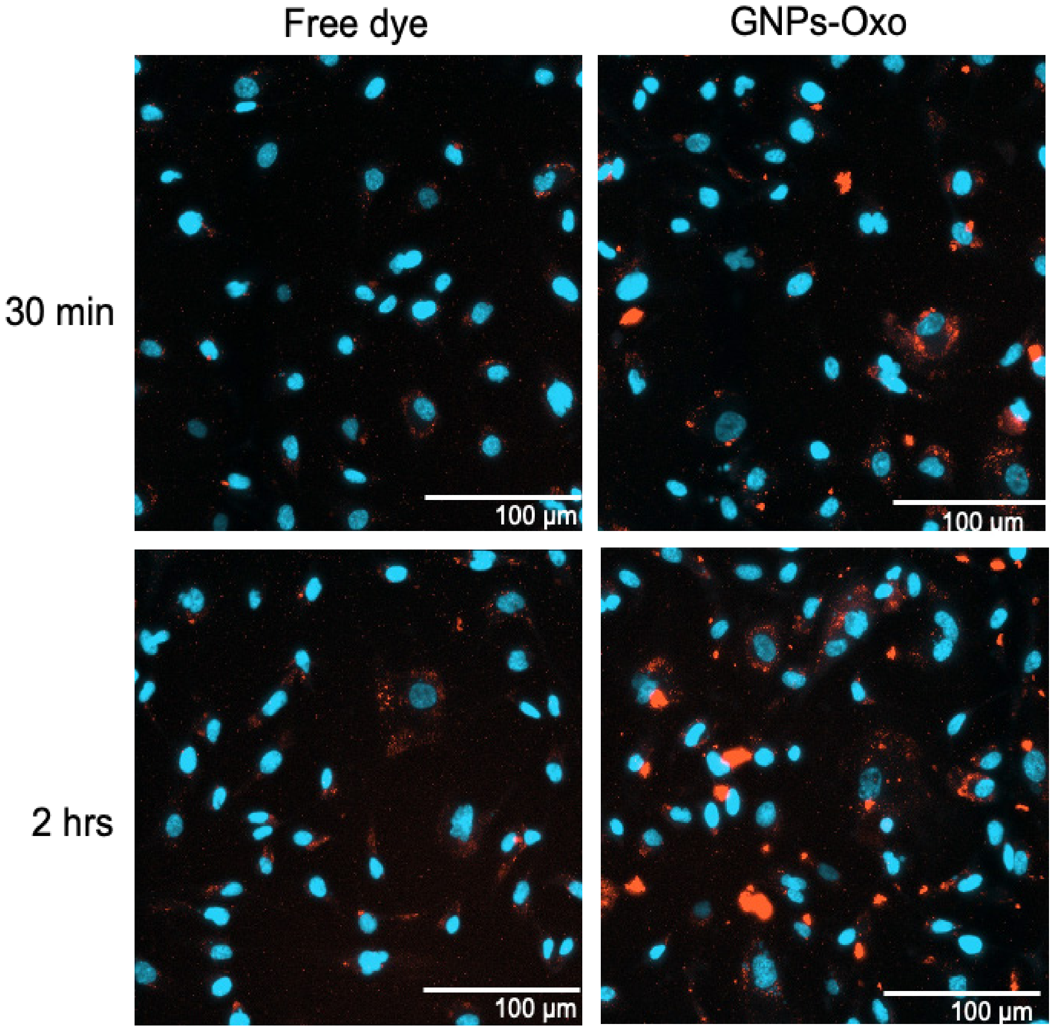

Figure S7 The distribution of nanoparticles conjugated with fluorescence dye (TAMRA) in HUVECs. Cells displayed clearer staining in the cytoplasm and even near nucleus 30 minutes post-treatment with GNPs-Oxo and much more staining intracellularly 2 hours post-treatment with GNPs-Oxo. Red and blue represent fluorescence dye (TAMRA) and DAPI staining, respectively. Scale bar: 100 µm.

Figure S8

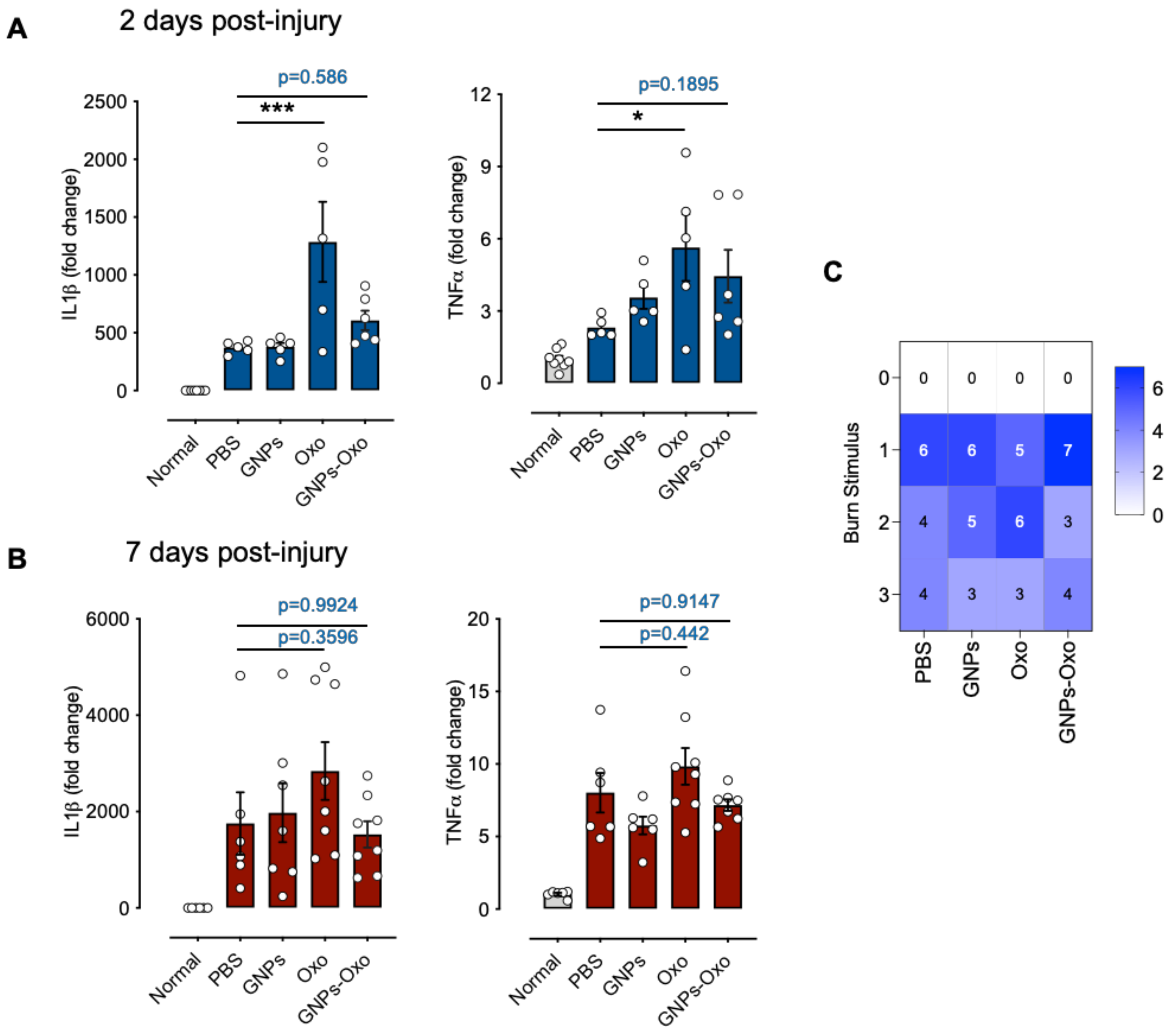

Figure S8 Gelatin nanoparticle-encapsulated 5Z-7-oxozeaenol reduces inflammatory responses developed in the mouse model of CoNV. (**A and B**) IL-1β and TNFα expression was significantly increased 2- and 7-days post-injury in mice, while GNP-Oxo treatment moderately reduced the IL-1β and TNFα expression (n = 5-8 corneas per treatment group). **(C)** Quantitative analysis of corneal blisters after chemical cauterization showed no difference among groups (see methods about the grading; n = 14 animals per treatment group from three experiments). Statistical analysis was conducted by one-way ANOVA and Tukey's multiple comparison test. *$P < 0.05$, ***$P < 0.001$.

Uncropped WB images
Figure 3A

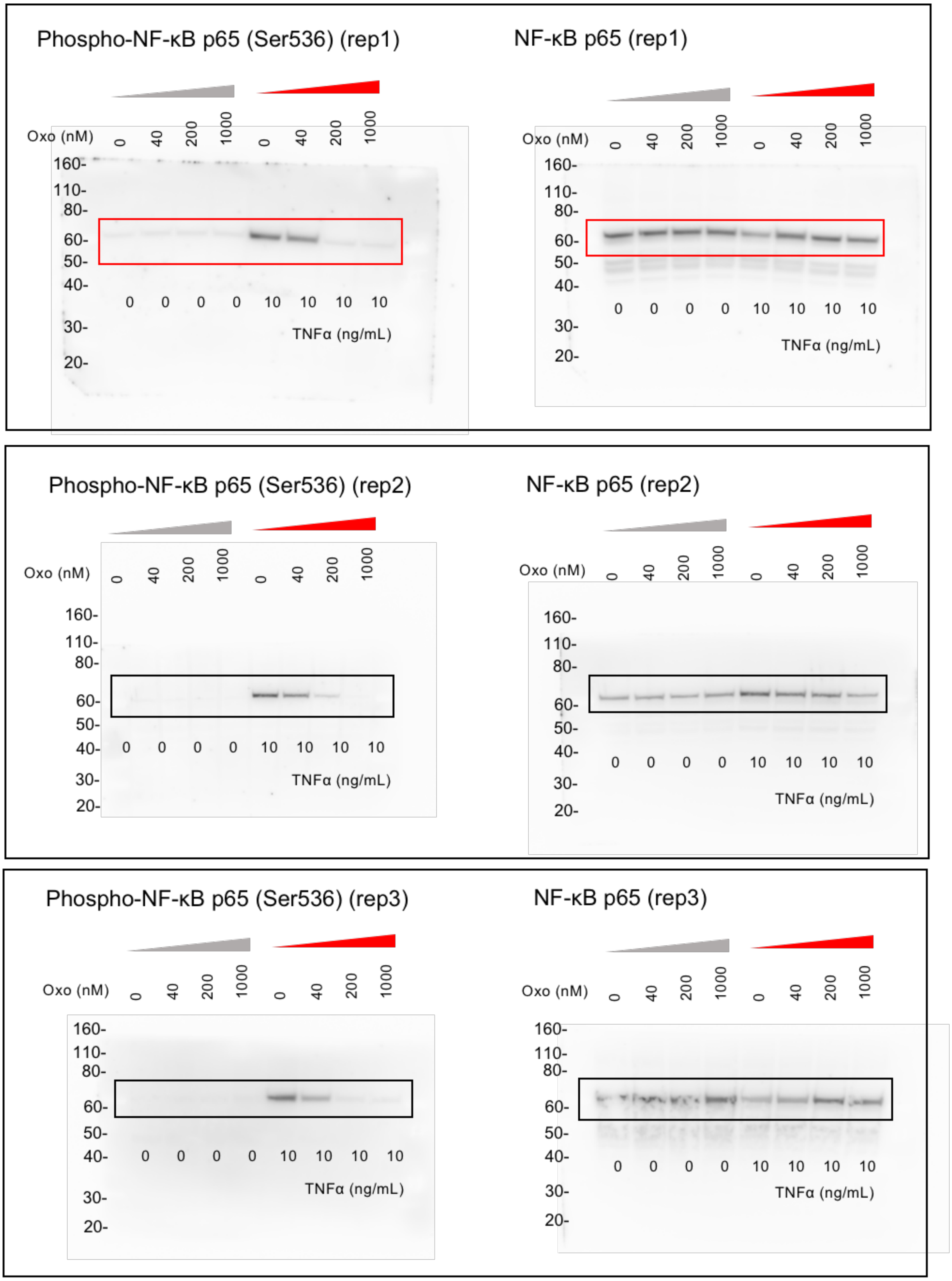

Figure 3A

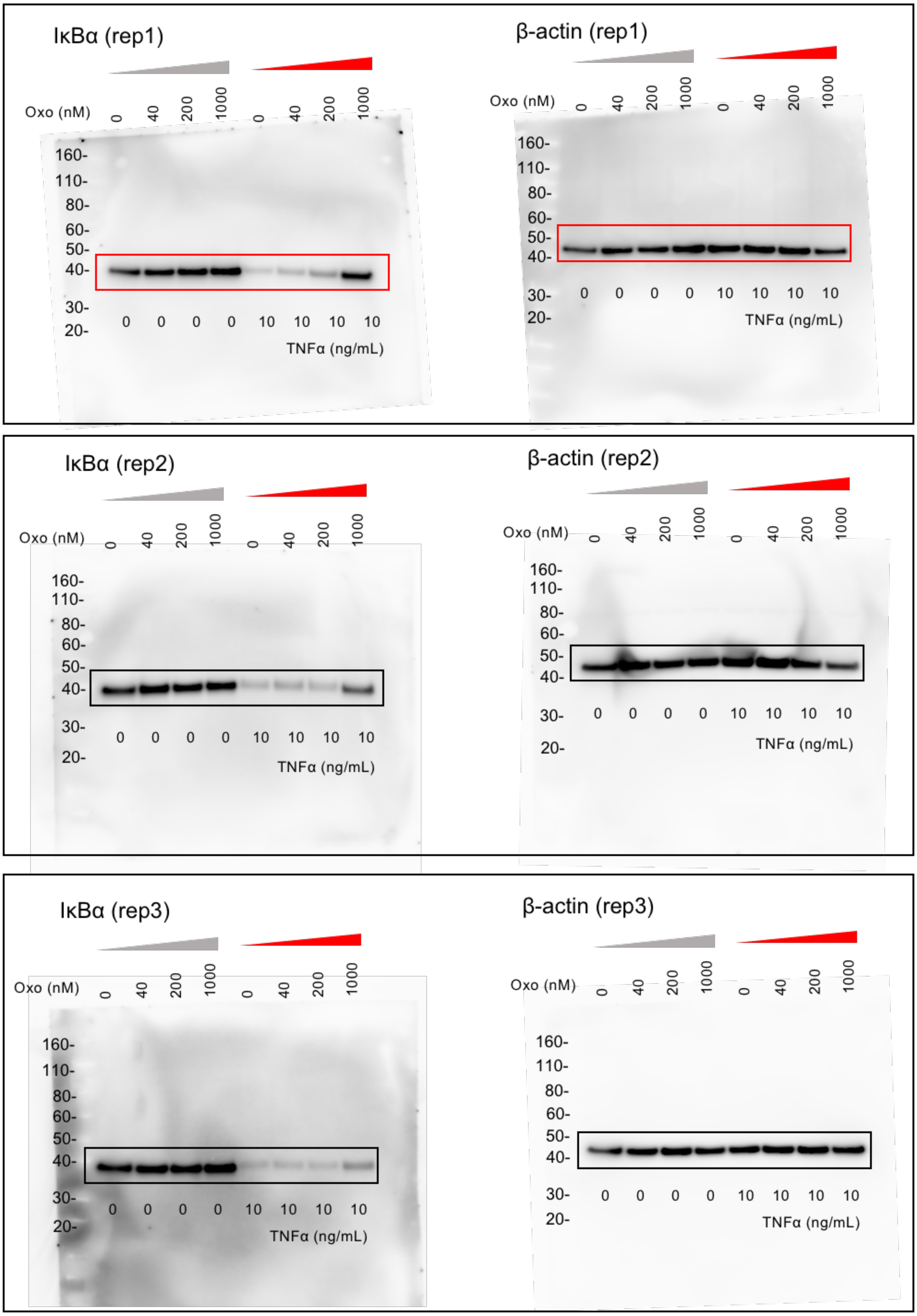
IκBα (rep1)
β-actin (rep1)
IκBα (rep2)
β-actin (rep2)
IκBα (rep3)
β-actin (rep3)
Oxo (nM)
0 40 200 1000 0 40 200 1000
160-
110-
80-
60-
50-
40-
30-
20-
0 0 0 0 10 10 10 10
TNFα (ng/mL)

Figure S5A

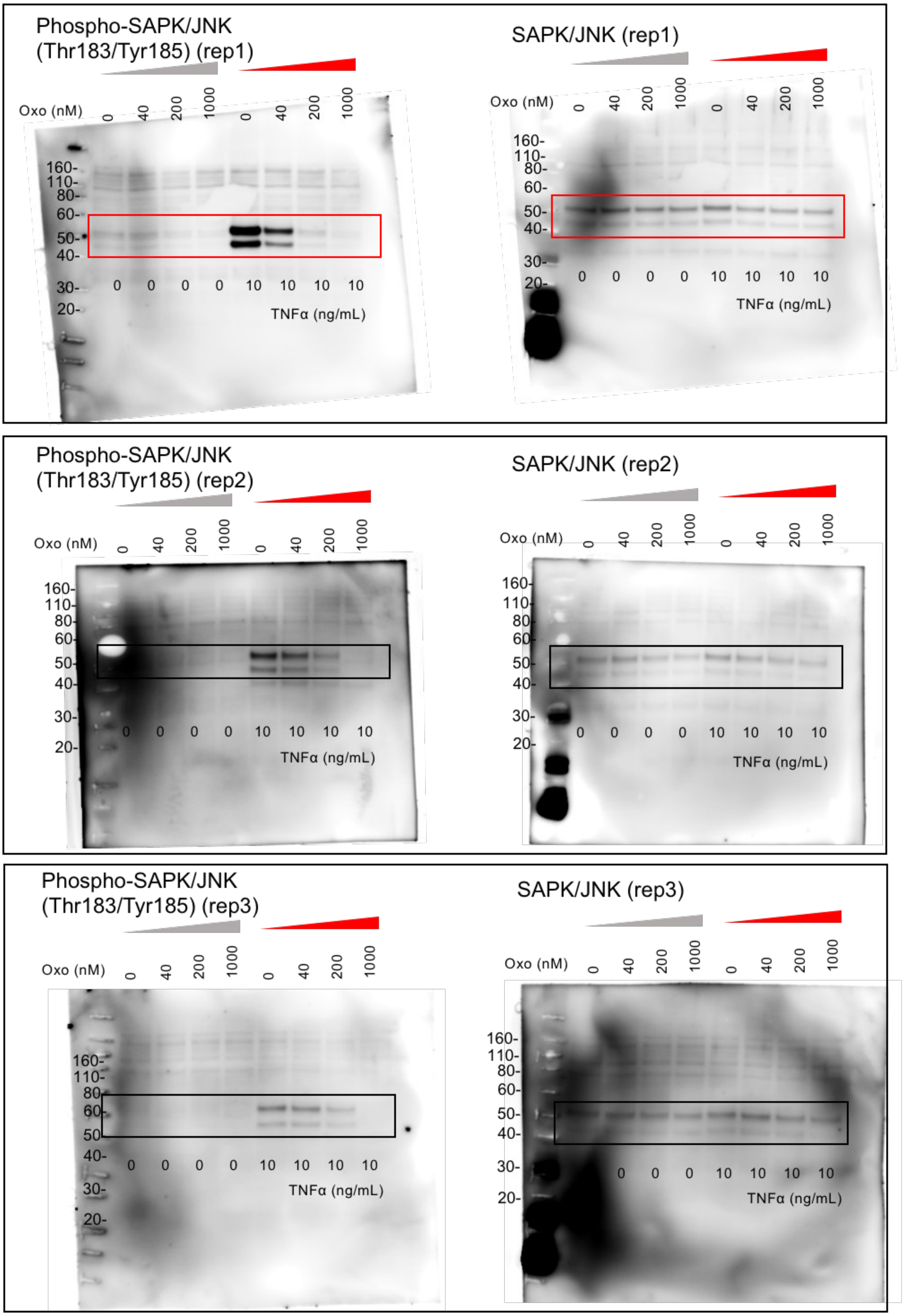
Phospho-SAPK/JNK (Thr183/Tyr185) (rep1)
SAPK/JNK (rep1)
Phospho-SAPK/JNK (Thr183/Tyr185) (rep2)
SAPK/JNK (rep2)
Phospho-SAPK/JNK (Thr183/Tyr185) (rep3)
SAPK/JNK (rep3)
Oxo (nM)
0 40 200 1000 0 40 200 1000
160- 110- 80- 60- 50- 40- 30- 20-
0 0 0 0 10 10 10 10
TNFα (ng/mL)

Figure S5A

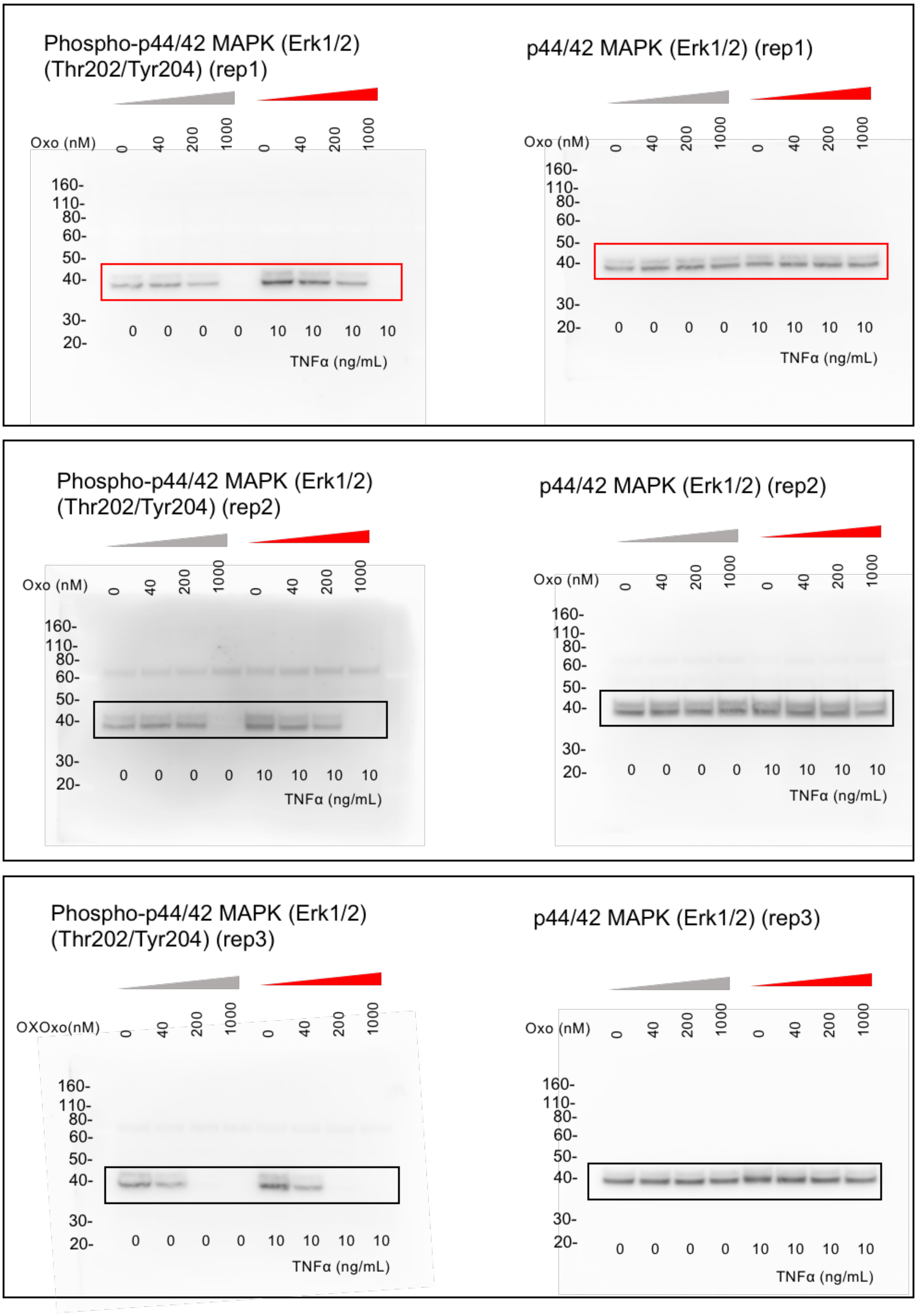

Phospho-p44/42 MAPK (Erk1/2) (Thr202/Tyr204) (rep1)
p44/42 MAPK (Erk1/2) (rep1)
Phospho-p44/42 MAPK (Erk1/2) (Thr202/Tyr204) (rep2)
p44/42 MAPK (Erk1/2) (rep2)
Phospho-p44/42 MAPK (Erk1/2) (Thr202/Tyr204) (rep3)
p44/42 MAPK (Erk1/2) (rep3)
Oxo (nM) 0 40 200 1000 0 40 200 1000
160- 110- 80- 60- 50- 40- 30- 20-
0 0 0 0 10 10 10 10
TNFα (ng/mL)
OXOxo(nM)

Figure S5A

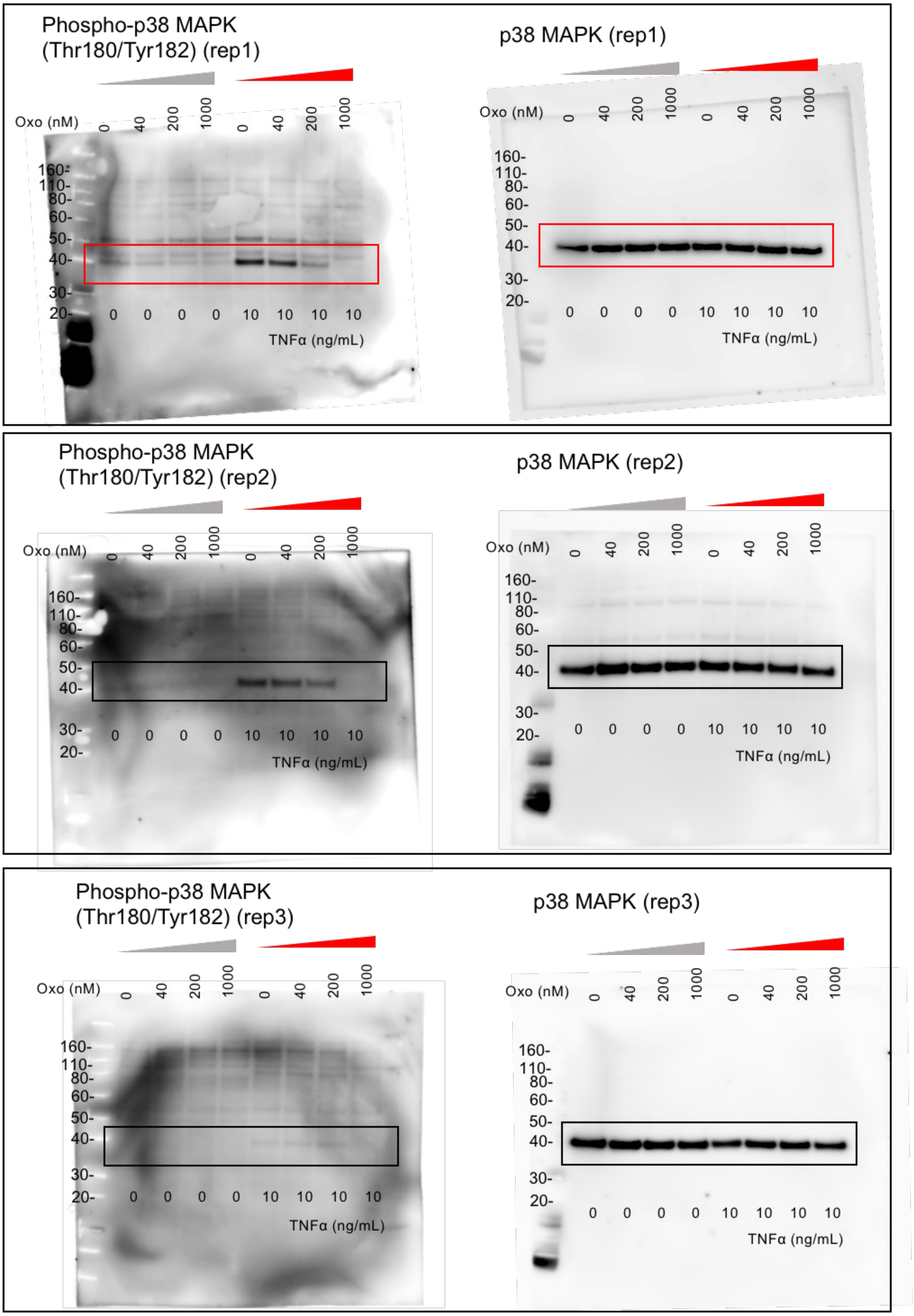

Phospho-p38 MAPK (Thr180/Tyr182) (rep1)
p38 MAPK (rep1)
Oxo (nM)
0 40 200 1000 0 40 200 1000
160-
110-
80-
60-
50-
40-
30-
20-
0 0 0 0 10 10 10 10
TNFα (ng/mL)
Phospho-p38 MAPK (Thr180/Tyr182) (rep2)
p38 MAPK (rep2)
Phospho-p38 MAPK (Thr180/Tyr182) (rep3)
p38 MAPK (rep3)
